# Identification of Ecological Corridors for Semi‐Aquatic Vertebrates: A Case of the Eurasian Otter in Northeast China

**DOI:** 10.1002/ece3.72429

**Published:** 2025-11-02

**Authors:** Qingyi Wang, Aihua Fu, Wendi Yang, Minhao Chen, Chao Zhang, Xiaofeng Luan

**Affiliations:** ^1^ School of Ecology and Nature Conservation Beijing Forestry University Beijing China; ^2^ Institute of Eco‐Environmental Research Guangxi Academy of Sciences Guangxi China; ^3^ National Park (Protected Area) Development Center, National Forestry and Grassland Administration Beijing China

**Keywords:** ecological corridors, Eurasian otter, Northeast China

## Abstract

As an indicator and flagship species of freshwater ecosystems, the Eurasian otter ( *Lutra lutra* ) plays a significant role in maintaining the connectivity and stability of ecosystems. However, compared with terrestrial and marine ecosystems, the conservation of freshwater ecosystems and species has not been sufficiently emphasized. In particular, research on the habitats and ecological corridors of semi‐aquatic vertebrates remains inadequate. This study employed species distribution models and corridor construction models to predict the distribution range of suitable habitats and identify the ecological corridors and key points for the Eurasian otter in Northeast China. The results showed that our models demonstrated good performance in species distribution model evaluation/validation, with average True Skill Statistic (TSS) and Area Under the Curve (AUC) values of 0.934 and 0.995 respectively. The model predicted that the suitable habitats of the Eurasian otter were mainly distributed along the rivers in the forested mountainous areas including the Daxing'anling Mountains, the Xiaoxing'anling Mountains, and the Changbai Mountains. Moreover, there were 39 core habitats, 42 ecological corridors, 78 pinch points and 19 barrier points identified. Among these, 11.92% of the suitable habitats, 25.83% of the core habitats and 18.66% of the ecological corridors were included in the scope of protected areas, but there were still protection gaps. In the future, anthropogenic disturbances in the Northeast China Forest Belt should be strictly controlled, and the integrated protection of forest and freshwater ecosystems should be strengthened so as to improve ecosystem and species connectivity.

## Graphical abstract

This study employed species distribution models and corridor construction models to predict the distribution range of suitable habitats and identify the ecological corridors and key points for the Eurasian otter in Northeast China. There were 39 core habitats, 42 ecological corridors, 78 pinch points and 19 barrier points identified. Among these, 11.92% of the suitable habitats, 25.83% of the core habitats and 18.66% of the ecological corridors were included in the scope of protected areas, but there were still protection gaps. In the future, anthropogenic disturbances in the Northeast China Forest Belt should be strictly controlled, and the integrated protection of forest and freshwater ecosystems should be strengthened so as to improve ecosystem and species connectivity.

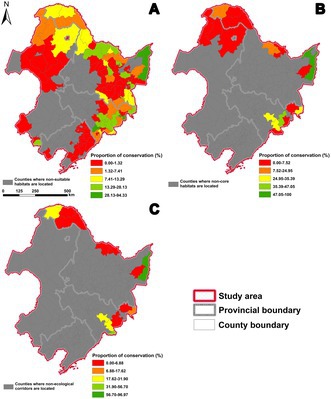

## 1 Introduction

Due to natural factors such as wildfires and floods, or human‐induced factors including land use changes, large and intact habitats have been fragmented into multiple smaller patches and may even be ultimately lost (Franklin et al. 2002). When habitat fragmentation occurs, the area, structure, and quality will change (Karsai and Kampis 2011), which in turn jointly affects the dynamics and persistence of species (Holland and Bennett 2010; Hovland et al. 1999), populations (Chen et al. 2019), and communities (Luther et al. 2020) at different temporal and spatial scales. Moreover, the interaction between habitat fragmentation and factors like human activities, climate change, and species interactions may further exacerbate the negative impacts of fragmentation (Ewers and Didham 2006).

Faced with the crisis of global species extinction caused by the fragmentation of habitats (Hanski et al. 2013), studies have shown that connecting habitat fragments is more conducive to biodiversity conservation (Karsai and Kampis 2011). As a method to enhance the connectivity between habitats, promote the communication among species individuals, and maintain the population sizes of animals and plants, protecting, restoring, and constructing ecological corridors to connect fragmented habitats has become a key strategy for biodiversity conservation (Reed 2004; Tewksbury et al. 2002). Compared with patches without corridor connections, corridors can increase the connectivity between habitat patches by approximately 50% (Gilbert‐Norton et al. 2010). Although some studies have indicated that corridors may not be significantly effective in maintaining biodiversity (Correa and Moura 2009; Falcy and Estades 2007; Öckinger and Smith 2008) and even bring negative impacts, such as the spread of diseases, the invasion of non‐native species, and increased probability of wildfires (Haddad et al. 2014; Resasco et al. 2014; Weldon 2006; Zhang et al. 2023), overall, the substantial positive impacts of promoting species dispersal and increasing species diversity (Brudvig et al. 2009; Damschen et al. 2006) outweigh the relatively minor and controllable negative impacts (Haddad et al. 2014; Resasco et al. 2023).

Currently, some studies have employed empirical research methods, including field observations, to identify ecological corridors for species such as the Blanding's turtle ( *Emydoidea blandingii* ) (Christensen and Chow‐Fraser 2014), the Blanchard's cricket frog ( *Acris blanchardi* ) (Badje et al. 2021), wood frog ( *Lithobates sylvaticus* ) (Anderson et al. 2015), jaguar ( *Panthera onca* ) (Caruso et al. 2023), and the African elephant ( *Loxodonta africana* ) (Chan et al. 2024). Empirical research methods can ensure the accuracy and effectiveness of corridor identification for different species. However, due to factors like the long duration required for data collection, it is difficult to apply the methods to the construction of large‐scale corridors (Shan et al. 2019).

With the development of landscape ecology, corridors have been incorporated into the patch‐corridor‐matrix model (Turner 2005). The methods for constructing ecological corridors have gradually matured, and a basic pattern for identifying large‐scale corridors through the process of “source identification–resistance surface construction–corridor extraction” has been formed (Li et al. 2023). The resistance model theory and circuit theory are widely used. The former posits that species need to overcome certain “resistances” or expend certain “costs” during migration and dispersal, and the pathways with the lowest accumulated resistance or cost are considered the optimal corridors (Knaapen et al. 1992). Based on this theory, some application tools, such as LandScape Corridors (Ribeiro et al. 2017) and Linkage Mapper (McRae et al. 2008), have been used for connectivity analysis. The latter, based on the random‐walk characteristics of species similar to electric currents, regards the landscape surface as a conductive surface. The higher the current density in the landscape, the greater the probability that species will pass through that area (Song and Qin 2016). Common software based on circuit theory includes CircuitScape and Omniscape (Dickson et al. 2019).

Nowadays, advanced corridor identification methods, particularly landscape connectivity modeling using Linkage Mapper and CircuitScape, have been successfully applied across diverse taxa, including mammals (Zhuo et al. 2022), birds (Chen et al. 2024; Sun et al. 2023), and plants (Zhang et al. 2024). Among the biological groups, large carnivores are more sensitive to habitat fragmentation and are flagship species that require focused protection, making them commonly regarded as a subject for connectivity research (Bhatt, Castley, Baral, and Chauvenet 2023). In addition to single‐species corridor research, a few studies have also considered multi‐species corridor identification, such as drawing corridors between protected areas to conserve plant diversity (Jewitt et al. 2017), optimizing the design of corridors between urban and suburban areas to enhance waterbird diversity (Yang et al. 2022), determining core habitats and migration routes for amphibians (Lee et al. 2022), and identifying large mammal corridors in mountainous regions (Meyer et al. 2020).

However, semi‐aquatic vertebrates like otters, minks, and beavers frequently move and disperse along dendritic aquatic ecosystems, posing challenges for identifying connectivity corridors (Carranza et al. 2012). Current studies have employed modeling approaches to analyze connectivity in species such as the bog turtle ( *Glyptemys muhlenbergii* ) (Travis et al. 2018), the Eurasian beaver ( *Castor fiber* ) (Falaschi et al. 2024; Serva et al. 2024), the Eurasian otter ( *Lutra lutra* ) (Leoncini et al. 2023; Van Looy et al. 2014), and the smooth‐coated otter ( *Lutrogale perspicillata* ) (Acharya et al. 2023). Nevertheless, research on habitats and ecological corridors for semi‐aquatic vertebrates remains insufficient overall. Freshwater ecosystems provide survival conditions for at least 6% of global species (Dudgeon et al. 2006), holding critical value for biodiversity and ecosystem conservation. For instance, rivers and riparian zones offer suitable habitats and migration pathways for aquatic, terrestrial, and semi‐aquatic animals (Sánchez‐Montoya et al. 2023). Amid global environmental changes, biodiversity across ecosystems is facing accelerated decline, with freshwater ecosystems likely under the greatest threat (Alahuhta et al. 2019; Jackson et al. 2016). Globally, over 20% of mammal species dependent on inland wetlands are threatened, 3% of which are critically endangered (Collen et al. 2014). Yet, compared to terrestrial and marine ecosystems, freshwater ecosystem conservation has received inadequate attention (Van Rees et al. 2021). Approximately 70% of upstream catchments in global river basins lack protected areas, while only 11.1% of river basins achieve comprehensive protection (Abell et al. 2017).

As a top predator, the Eurasian otter ( *Lutra lutra* ) serves as both an indicator and flagship species for freshwater ecosystems (Zhang et al. 2018). It primarily inhabits rivers and riparian zones, preying on fish and amphibians (Fu et al. 2024; Zhan et al. 2023). However, due to river pollution and human persecution, Eurasian otter populations have drastically declined (Loy et al. 2022, accessed on 24 February 2025). Studies indicate that this species, once widely distributed across China (Li and Chan 2018), now has potential habitats concentrated mainly in the southeastern Qinghai–Tibet Plateau and northeastern China, followed by the Qinling Mountains in Shaanxi Province, hilly areas in southeastern Guizhou Province, and sparse patches in southeastern regions (Zhang et al. 2018). Internationally, the Eurasian otter is classified as Near Threatened (NT) by the International Union for Conservation of Nature (IUCN) (Loy et al. 2022, accessed on 24 February 2025) and listed in the Convention on International Trade in Endangered Species of Wild Fauna and Flora (CITES) Appendix I (CITES 2024, accessed on 24 February 2025). In China, it is designated as a second‐class protected animal (National Forestry and Grassland Administration 2021, accessed on 24 February 2025).

Current research in China focuses mainly on its genetic diversity (Jang‐Liaw et al. 2023; Li, Yeh, et al. 2024; Zheng et al. 2024), population size (Yang et al. 2023), habitat preferences and distribution (Chen, Zhang, Fu, et al. 2023; Chen, Zhang, Wang, et al. 2023; Fung et al. 2024; Hui and Chan 2024; Wang et al. 2021; Zhang, Chen, et al. 2022; Zhang et al. 2016), diet (Dou et al. 2023; Fu et al. 2024; Wang et al. 2024; Wang, Wang, et al. 2022; Zhan et al. 2023; Zhao et al. 2022), activity patterns (Han et al. 2021), and public attitudes (McMillan et al. 2019). However, studies on ecological corridors for semi‐aquatic vertebrates like otters in China remain nascent. Amid urgent demands for freshwater ecosystem conservation, measures to protect suitable habitats for Eurasian otters and remove potential barriers along their movement pathways could advance the resolution of freshwater ecosystem and species conservation challenges (Cianfrani et al. 2011).

Therefore, to address these knowledge gaps, this study integrated species distribution models and corridor construction models to investigate the spatial ecology of the Eurasian otter in Northeast China. We aimed to achieve the following specific objectives: (1) to predict the spatial distribution of suitable habitats for the Eurasian otter; (2) to delineate core habitat patches, potential ecological corridors, and critical nodes including pinch points and barrier points within the landscape; and (3) to assess the degree to which these key areas are covered by the existing network of protected areas, thereby identifying conservation priorities.

## 2 Materials and Methods

### 2.1 Study Area

**FIGURE 1:**
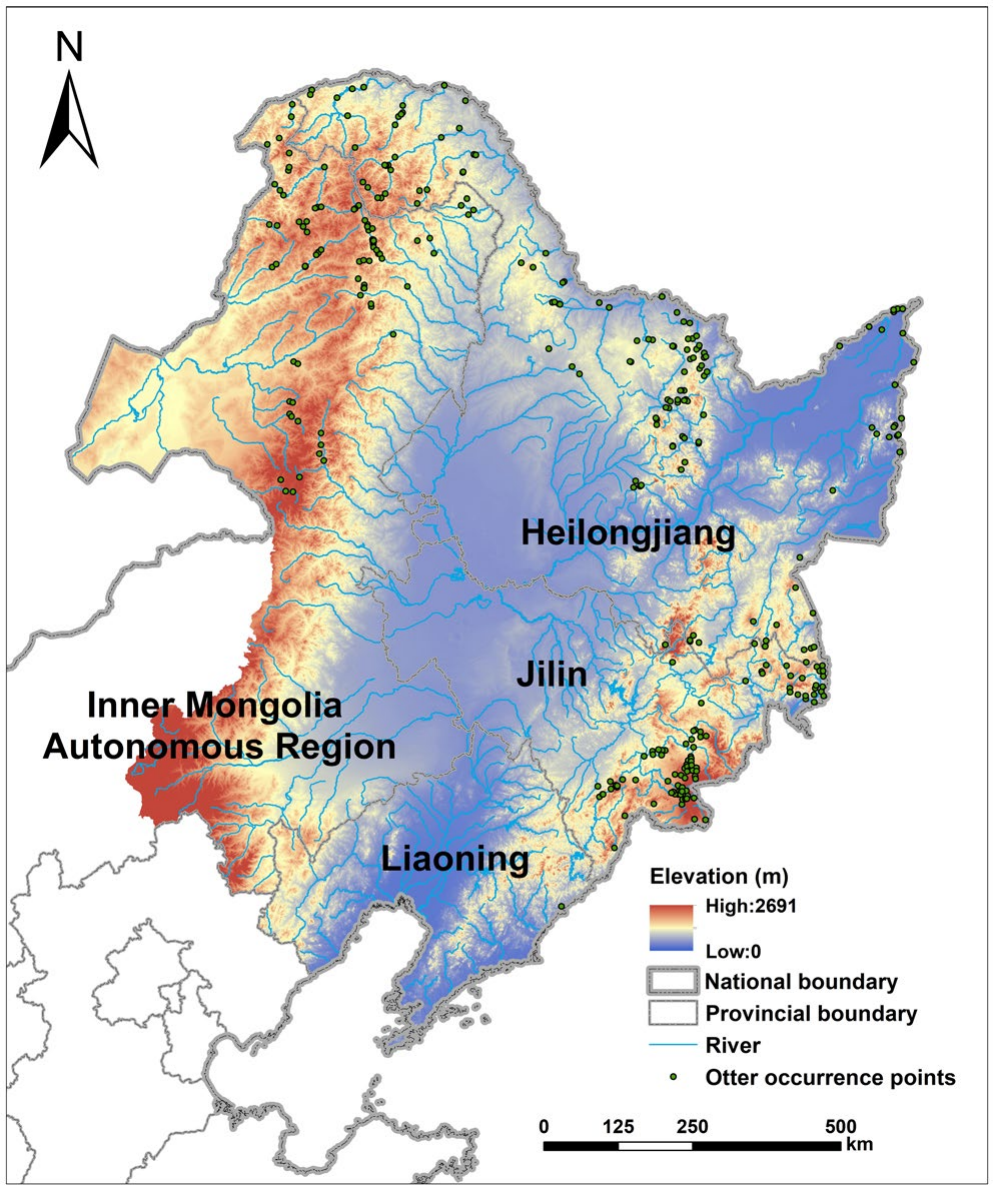
Study area and 356 collected occurrence points of Eurasian otters.

Northeast China (38°42′ N–53°55′ N, 115°52′ E–135°09′ E) is located in the eastern part of the Eurasian continent. It is bordered by North Korea to the east, the Bohai Sea to the south, Mongolia to the west, and Russia to the north. The region includes Heilongjiang Province, Jilin Province, Liaoning Province, and the northeastern part of Inner Mongolia Autonomous Region, specifically including Hulunbuir City, Xing'an League, Tongliao City, and Chifeng City. In total, it encompasses 40 prefecture‐level administrative regions and 322 county‐level administrative regions. With a vast territory and a total area of approximately 1.25 million km^2^, it accounts for 13.01% of China's total land area. The study area has an elevation ranging from 0 to 2691 m and features complex and diverse topography. The central, southern, and eastern parts are respectively characterized by the Songnen Plain, Liaohe Plain, and Sanjiang Plain. The region is divided by mountain ranges such as the Daxing'anling Mountains, the Xiaoxing'anling Mountains, and the Changbai Mountains. The main rivers include the Heilongjiang River, Songhua River, Nen River, and Liao River (Figure 1). The region has a temperate continental climate with distinct seasons, cold winters, and hot summers (Song et al. 2021). Northeast China is rich in plant resources, with the largest area of natural forests in the country (Yu et al. 2011). These forests provide important habitats for several rare and endangered wildlife species, such as the Siberian tiger (*Panthera tigris amurensis*), red‐crowned crane ( *Grus japonensis* ), and musk deer ( *Moschus moschiferus* ) (Wan et al. 2018). In the national ecological functional zoning (Chen et al. 2022), the region includes four key biodiversity conservation areas: the Xiaoxing'anling Mountains Biodiversity Conservation Area, Sanjiang Plain Wetland Biodiversity Conservation Area, Songnen Plain Biodiversity Conservation and Flood Regulation Area, and Liaohe Delta Wetland Biodiversity Conservation Area. These areas cover a total of 129,200.00 km^2^ and provide significant support for biodiversity conservation.

### 2.2 Data Collection and Processing

The distribution data of Eurasian otters were collected from three sources: (1) Literature and reports from China National Knowledge Infrastructure (CNKI) and Google Scholar; (2) Open biodiversity databases, including the Global Biodiversity Information Facility (GBIF, https://www.gbif.org/), VertNet (https://vertnet.org/), Integrated Digitized Biocollections (iDigBio, https://www.idigbio.org/), and iNaturalist (https://www.inaturalist.org/); notably, GBIF already incorporates research‐grade observations from iNaturalist; (3) Field data obtained through interview surveys, questionnaire surveys, and scat collection in Northeast China from 2016 to 2020. This research involved human participants through interviews and questionnaires. In accordance with the national guidelines and the principles outlined in the Helsinki Declaration, all participants were informed about the study's purpose and their anonymous, voluntary participation. Verbal informed consent was obtained from all participants before their involvement, and no personally identifiable information was recorded. A total of 356 occurrence points of Eurasian otters in Northeast China from 2000 to 2024 were compiled (Figure 1). To mitigate the negative impacts of spatial autocorrelation on model simulations, points within 3 km were thinned based on the minimum home range of otters, retaining only one randomly selected point within each 3 km radius (Chen, Zhang, Fu, et al. 2023; Chen, Zhang, Wang, et al. 2023; Zhang, Chen, et al. 2022). Ultimately, 269 occurrence points were used for species distribution modeling (Table S1). The final modeling dataset comprised records solely from literature and reports (Source 1) and field surveys (Source 3), as records from the biodiversity databases (Source 2) were thinned out during this process due to spatial proximity.

Species distribution ranges are closely related to the environment (Gaston 2009; Murray and Conner 2009). Therefore, the selection of environmental variables is fundamental to constructing accurate species distribution models. In this study, based on the biological characteristics of the Eurasian otter and its habitat requirements (Barbosa et al. 2001; Chen, Zhang, Wang, et al. 2023; Macdonald and Mason 1983; Zhang, Chen, et al. 2022), 21 environmental variables were selected across five major categories (Table A1). The slope, aspect, and ruggedness (Sappington et al. 2007) were derived from the Digital Elevation Model (DEM). Distances to railways, roads, residential areas, dams, lakes, reservoirs, and rivers were generated using the Euclidean Distance tool in the Geographic Information System (GIS). The density of cultivated land and forest land was extracted from land use type data by selecting corresponding raster layers and then generated using the Kernel Density Analysis tool in GIS. All environmental variable data were unified into the same geographic coordinate system (Xian 1980) with a resolution of 1 km.

Multicollinearity among environmental variables can lead to overfitting in species distribution models (Pradhan 2016). This study selected 15 environmental variables with a Variance Inflation Factor (VIF < 10) (Naimi et al. 2014) for the final model construction (Table 1).

**TABLE 1:** Environmental variables used for species distribution model with their VIF values.

Environmental variables	VIF value
bio15	1.37
bio18	2.48
Elevation	2.21
Slope	2.21
Aspect	1.01
Ruggedness	1.89
Population density	1.02
Distance from railways	1.39
Distance from roads	1.22
Distance from residential areas	1.73
Distance from dams	2.72
Density of cultivated land	3.75
Density of forest land	5.28
Distance from water area	2.44
Distance from rivers	1.17

### 2.3 Species Distribution Model Building

Species Distribution Models (SDMs) are powerful tools for predicting species distributions across spatial and temporal scales (Guisan et al. 2017), playing a critical role in understanding ecological processes and informing conservation planning for flora and fauna (Dormann et al. 2012). With the rapid advancement of SDMs, diverse algorithms originally developed for other purposes—such as Random Forest (RF), Generalized Linear Model (GLM), and Maximum Entropy (MAXENT)—have been successfully adopted and applied within the field. While each model has distinct strengths and limitations (Elith et al. 2006; Li and Wang 2013), reliance on a single algorithm may introduce uncertainty in predictive outcomes (Qiao et al. 2019). As one of the R packages providing an integrated platform for running SDMs, biomod2 enables the concurrent execution of over a dozen modeling approaches, avoiding overreliance on any single method, and synthesizes ensemble predictions to enhance model robustness and transferability (Araújo and New 2007; Breiner et al. 2015). Nowadays, biomod2 is widely applied in conservation research and management practices, including invasive species management (Ángel‐Vallejo et al. 2024) and wildlife protection (Li, Jiang, et al. 2024; Zhang and Wang 2023).

Based on the biomod2 package (Version: 4.2–6‐1) in R (Version: 4.4.1), a total of 11 algorithms were used to construct single models initially, namely Artificial Neural Network (ANN), Classification Tree Analysis (CTA), Flexible Discriminant Analysis (FDA), Generalized Additive Model (GAM), Generalized Boosting Model (GBM), Generalized Linear Model (GLM), Multiple Adaptive Regression Splines (MARS), Maximum Entropy (MAXENT), Random Forest (RF), Surface Range Envelop (SRE/BIOCLIM), and eXtreme Gradient Boosting Training (XGBOOST). Since the absence points of the Eurasian otter could not be obtained, we randomly selected pseudo‐absence points with the same number as the presence points in three independent replicates (Huang et al. 2023). We employed a modeling approach where the total weight of pseudo‐absence points was set equal to the total weight of presence points (Liu et al. 2019).

**FIGURE 2:**
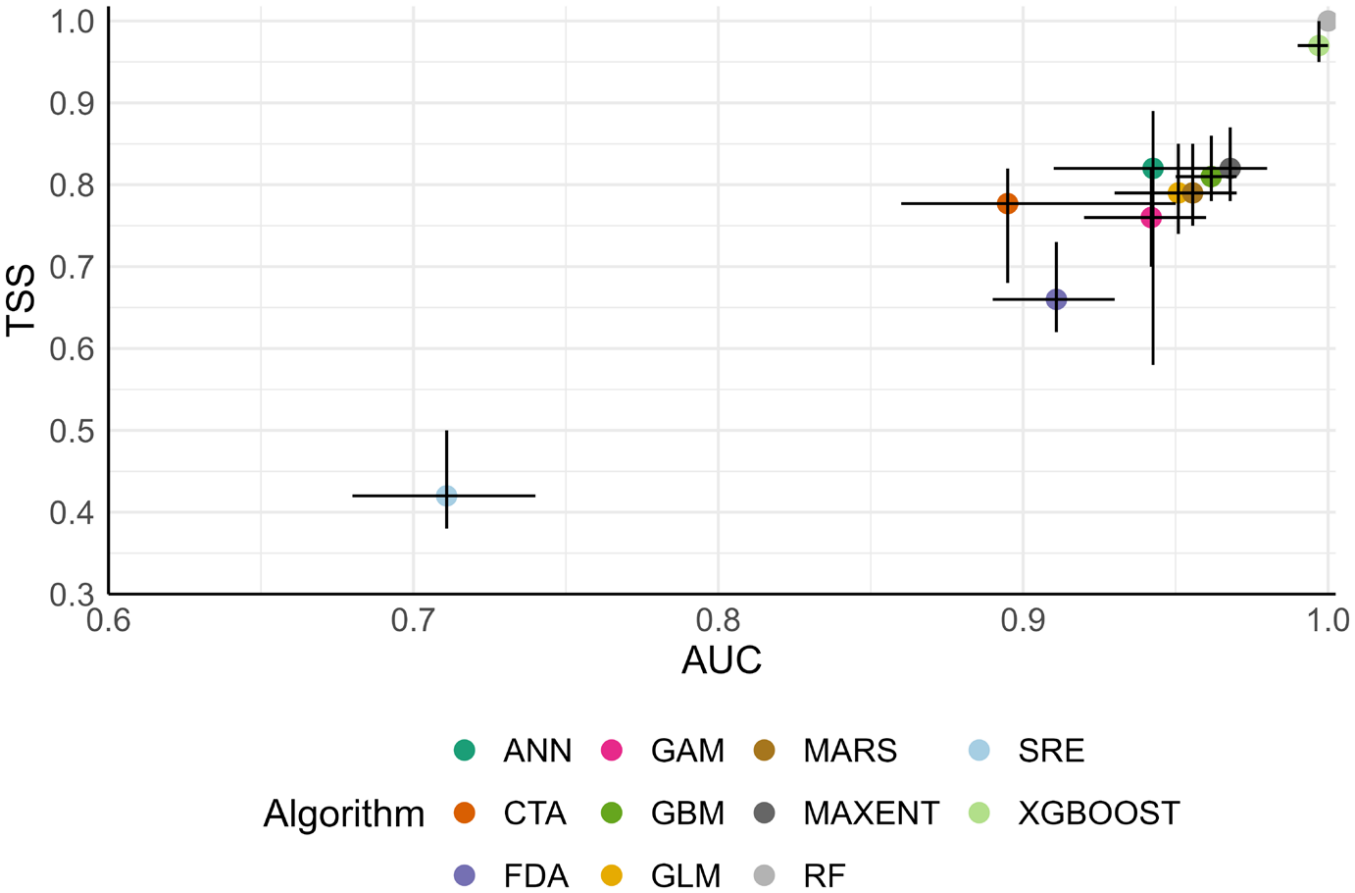
The optimal modeling algorithm selected by using True Skill Statistics (TSS) and Area Under the Curve (AUC). Dots with different colors represent the average value of different algorithms after ten operations; error lines indicate maximum and minimum values. Models (RF and XGBOOST) with TSS > 0.85 and AUC > 0.90 were selected.

To evaluate the models, we selected 80% of the data as the training sample and 20% as the validation sample, and conducted 10 iterative operations. In this study, models with TSS > 0.85 (Li, Luo, et al. 2024) and AUC > 0.90 (Huang et al. 2023; Zhao et al. 2021) were selected, namely RF and XGBOOST (Figure 2). The final ensemble model was constructed using a weighted averaging approach, where the weights were based on TSS, and the importance of environmental variables was assessed through the variables_importance function in the biomod2 package. Finally, the continuous habitat suitability was converted to binary outputs by maximizing the TSS score of the output binary prediction.

### 2.4 Connectivity Analysis

#### 2.4.1 Source Identification

Source refers to the starting points for species migration and dispersal. Different studies employ various methods to identify sources, including designating protected areas where the target species are distributed (Giliba et al. 2023; Wu et al. 2024; Zhang, Hu, et al. 2022), or using high‐suitability distribution zones predicted by SDMs (Liu et al. 2023; Yu et al. 2024) as sources. However, directly regarding protected areas or specific ecological functional zones, or high‐suitability distribution zones as sources, can be highly policy‐driven and subjective (Han et al. 2025). Some studies employ comprehensive evaluation approaches, such as integrating habitat suitability with environmental quality (Ma and You 2022; Yang et al. 2024), to refine the scope of source areas. Moreover, tools like the Morphological Spatial Pattern Analysis (MSPA) tool in the Guidos Toolbox developed by the Joint Research Centre (JRC) of the European Commission (Chen et al. 2024), and the Core Mapper tool in the Gnarly Landscape Utility Tool (Acharya et al. 2023), can identify core areas. Based on the habitat suitability of the Eurasian otter, we utilized the Core Mapper tool (Shirk and McRae 2013) to calculate the core habitats. Using the minimum home range area of the Eurasian otter (approximately 28 km^2^) as a constraint, we identified 39 core habitats as the sources for connectivity analysis.

#### 2.4.2 Resistance Surface Construction

A resistance surface quantifies the obstacles a species encounters when moving across heterogeneous landscapes, such as rugged terrain or human‐dominated land use types (Yu et al. 2022). Higher resistance values indicate greater difficulty for the species to disperse or migrate through the area. Two primary methods exist for constructing resistance surfaces: (1) expert‐based assignment of resistance values to landscape features (Chen et al. 2024; Yang et al. 2024), and (2) conversion of habitat suitability layers into resistance surfaces (Giliba et al. 2023; Hofman et al. 2018). Studies demonstrate that corridors identified using habitat quality‐based resistance surfaces are more ecologically accurate than those derived from expert opinion (Zhou et al. 2024).

Accordingly, we rescaled habitat suitability values to a 0–1 range and transformed the habitat suitability layer into a resistance surface using a negative exponential function (Trainor et al. 2013). The formula is expressed as:

$$R=100-99\times \frac{1-e^{-\mathrm{ch}}}{1-e^{-c}}$$

where *R* represents the resistance value of a grid cell, *h* denotes the habitat suitability value, and *c* is a curvature constant governing the shape of the negative exponential function. Based on empirical evidence that intermediate values of *c* optimize performance (Keeley et al. 2016), we set *c* = 4.

#### 2.4.3 Corridor Extraction and Node Recognition

In this study, the Linkage Mapper tool was employed to identify ecological corridors by importing source and resistance surfaces. Based on the home range and dispersal distances of the Eurasian otter (Durbin 1993; White et al. 2003), the threshold for cost‐weighted distance was set at 10 km, and the maximum Euclidean distance for corridors was set at 40 km.

Ecological pinch points are areas with high probabilities of species passage or regions that cannot be substituted during migration (McRae et al. 2008). We used the Linkage Mapper toolbox, which employed the stand‐alone CircuitScape software, to identify ecological pinch points within corridors. Specifically, CircuitScape was run in “all‐to‐one” mode for this analysis. Ecological barrier points refer to regions that pose significant obstacles to species migration (McRae et al. 2012). The Barrier Mapper module in the Linkage Mapper toolbox was used to identify these points. The “Maximum” mode was selected for iterative calculations, the minimum search radius was set at 1 km, and the maximum search radius was also set at 3 km, with a step size of 0.5 km.

To explain the underlying reasons behind identified barrier points, we generated a set of randomly selected points (*n* = number of identified barrier points) located strictly within the potential ecological corridors. Then we extracted values for key environmental factors known to be major contributors to landscape resistance for our focal species based on our initial resistance surface, and performed non‐parametric Mann–Whitney *U* tests to compare the distribution of each environmental variable's values between the identified barrier points and the random points within the corridors.

### 2.5 Identification of Priority Conservation Areas

To evaluate the quality of different corridors, we used the ratios of cost‐weighted distance to Euclidean distance (CWD: EucD), cost‐weighted distance to least‐cost path (CWD: LCP), and cost‐weighted distance to effective resistance (CWD: Effres) for assessment (Dutta et al. 2016). A higher value of CWD: EucD indicates a higher movement cost for species between sources; CWD: LCP represents the average resistance encountered by species during their spread; a higher value of CWD: Effres means that the corridor is more robust, i.e., there are more alternative corridors (Bhatt, Castley, Sims‐Castley, et al. 2023). Moreover, we performed an overlay analysis of the scope of the protected areas (https://www.resdc.cn/, accessed on 24 February 2025) with the obtained suitable habitat and corridor layers of the Eurasian otter to identify conservation gaps and priority conservation areas.

## 3 Results

### 3.1 Model Performance and Importance of Variables

In the prediction of suitable habitats for the Eurasian otter, an ensemble model combining RF and XGBOOST achieved high predictive accuracy, with a mean TSS of 0.934 and a mean AUC of 0.995. The four most critical environmental variables influencing Eurasian otter distribution, ranked by importance, were: distance from rivers, density of forest land, density of cultivated land, and precipitation seasonality (Table 2).

**TABLE 2:** Importance of variables.

Sort by importance	Variables	Importance
1	Distance from rivers	0.276
2	Density of forest land	0.072
3	Density of cultivated land	0.052
4	bio15	0.046
5	bio18	0.014
6	Ruggedness	0.005
7	Distance from roads	0.005
8	Distance from water area	0.004
9	Distance from dams	0.004
10	Slope	0.003
11	Population density	0.003
12	Elevation	0.002
13	Aspect	0.002
14	Distance from railways	0.002
15	Distance from residential areas	0.002

### 3.2 Suitable Habitats

**FIGURE 3:**
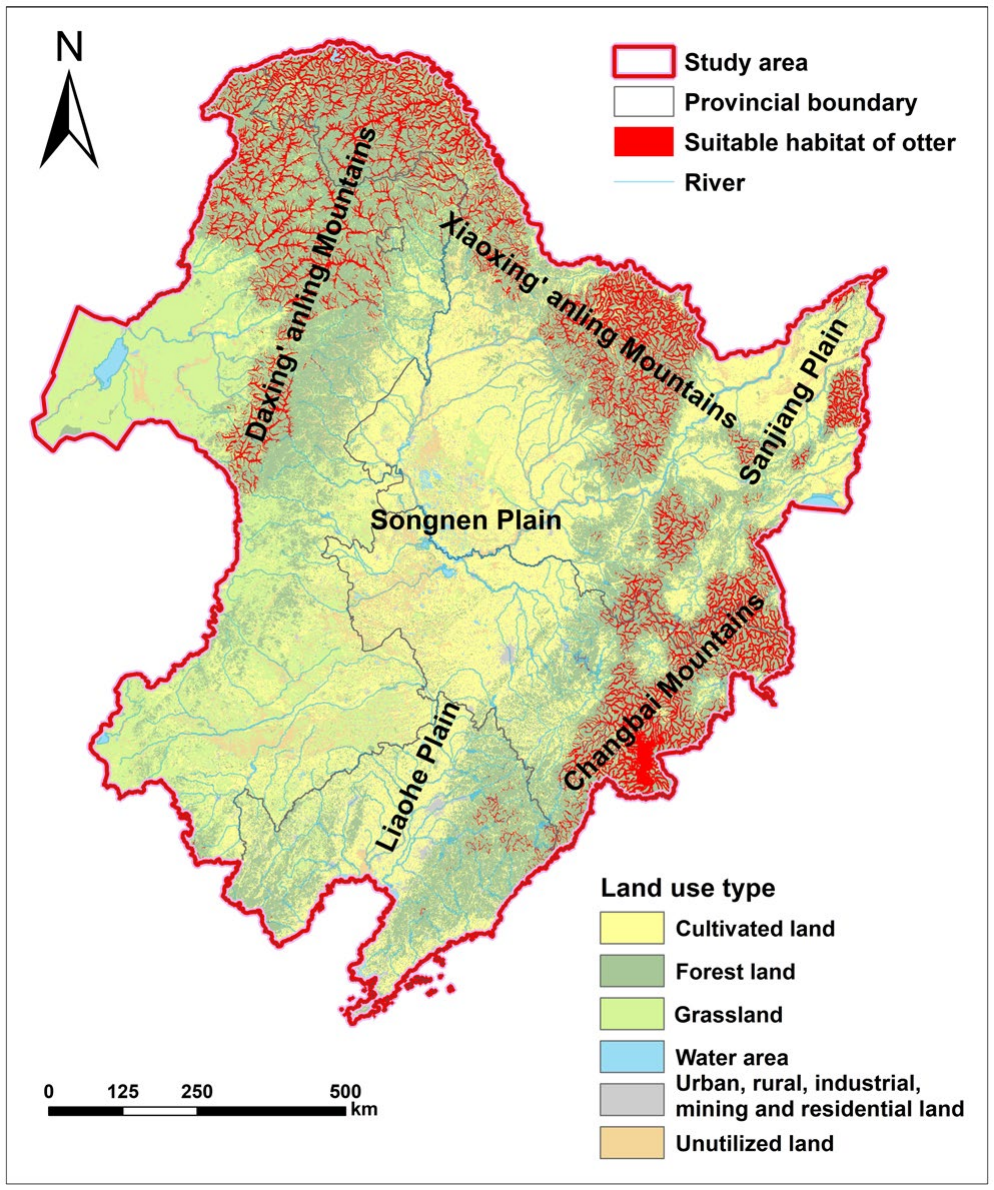
Potential suitable habitat distribution of the Eurasian otter. These potential suitable habitats were primarily distributed in a dendritic pattern along rivers in the Daxing'anling Mountains, the Xiaoxing'anling Mountains, and the Changbai Mountains.

The results of the SDMs predicted that the area of potentially suitable habitats for the Eurasian otter was 109,400.00 km^2^, accounting for 8.76% of the study area. These suitable habitats were primarily distributed in a dendritic pattern along rivers in the Daxing'anling Mountains, the Xiaoxing'anling Mountains, and the Changbai Mountains (Figure 3). Of the suitable habitats, 69.41% were located in forest land, followed by grassland, with 58.37% specifically found in woodland within forest land (Table A2).

In the study area, over half of the suitable habitats were distributed in Heilongjiang Province (53.58%), followed by Inner Mongolia Autonomous Region (23.66%), Jilin Province (21.50%), and Liaoning Province (1.26%). From a prefecture‐level perspective, the top three regions with the largest suitable habitat areas for the Eurasian otter were Hulunbuir City in Inner Mongolia Autonomous Region, the Daxing'anling Mountains area in Heilongjiang Province, and Yanbian Korean Autonomous Prefecture in Jilin Province, which together accounted for 52.43% of the total suitable habitat area. From a county‐level perspective, the top three counties with the largest suitable habitat areas were Huma County in the Daxing'anling Mountains area of Heilongjiang Province, Oroqen Autonomous Banner in Hulunbuir City of Inner Mongolia Autonomous Region, and Mohe City in the Daxing'anling Mountains area of Heilongjiang Province (Table A3). These three counties were adjacent to each other and were all located in the northern part of the study area.

### 3.3 Corridors and Nodes

The Eurasian otter's core habitats were concentrated in 39 areas, totaling 5006.00 km^2^, which accounted for 4.58% of its suitable habitats. Forest land (86.03%) represented the primary land use type for core habitat distribution (Table A2). Nearly 80% of the core habitats were located in Jilin Province, with Baishan City hosting the largest core habitat area (2024.83 km^2^, 40.45% of the total core habitat area; Table A4). Due to the species' limited dispersal capacity, five isolated core habitats remained unconnected. These were distributed in Erguna City, Genhe City, and the Oroqen Autonomous Banner (Hulunbuir City, Inner Mongolia Autonomous Region), as well as Fenglin County (Yichun City, Heilongjiang Province) and Tongjiang City (Jiamusi City, Heilongjiang Province), covering a total area of 188.00 km^2^.

Thirty‐four core habitats were ultimately connected by corridors, and forty‐two corridors were identified, with a total length of 804.77 km (Table 3). Among these, 75.14% of the corridors overlapped with forest land, followed by grassland (Table A2). Additionally, 33.48%, 13.26%, and 11.28% of the corridors were located in Wangqing County and Hunchun City (Yanbian Korean Autonomous Prefecture, Jilin Province) and Tahe County (Daxing'anling Mountains area, Heilongjiang Province), respectively (Table A5). Corridor 18, the longest corridor (55.46 km), was situated in Wangqing County (Yanbian Korean Autonomous Prefecture, Jilin Province). Among the identified corridors, several exhibited characteristics favorable for Eurasian otter movement: Corridors 8 (Xunke County, Heihe City, Heilongjiang Province), 9 (spanning Xunke County, Jiayin County, and Tangwang County in Heilongjiang Province), 20 (Wangqing County, Yanbian Korean Autonomous Prefecture, Jilin Province), and 32 (Huadian City, Jilin City, Jilin Province) demonstrated particularly low movement costs and minimal dispersal resistance. Corridor 37, located in Fusong County (Baishan City, Jilin Province), exhibited the highest robustness, indicating greater potential dispersal pathways and connectivity between the linked core habitats (Table 3). These specific characteristics (low movement cost, low dispersal resistance, high robustness) observed in certain corridors suggest their potential effectiveness in facilitating Eurasian otter migration and dispersal within the network.

**TABLE 3:** Ecological corridor quality assessment.

Code	From_Core	To_Core	Length (km)	CWD: EucD	CWD: LCP	CWD: Effres
1	1	2	12.49	3.36	2.44	4.76
2	3	4	44.38	2.36	2.00	5.78
3	3	5	25.31	3.72	2.19	6.01
4	4	5	38.14	3.22	1.92	5.16
5	4	6	3.83	3.46	2.02	5.39
6	5	7	2.41	3.77	1.56	3.95
7	11	12	17.31	2.82	1.79	4.79
8	11	14	19.31	**2.10**	**1.72**	5.22
9	12	13	10.41	**1.82**	**1.40**	5.41
10	12	14	6.41	3.01	2.35	6.23
11	13	14	2.83	5.73	2.87	9.88
12	17	18	39.21	2.23	1.91	7.78
13	17	19	16.66	2.17	1.81	4.52
14	18	19	22.49	2.32	1.99	5.46
15	18	20	17.07	2.36	1.93	4.63
16	19	20	14.90	2.31	1.89	4.74
17	21	22	52.63	4.21	1.76	5.84
18	21	25	**55.46**	3.75	2.07	8.79
19	22	23	17.49	2.53	2.02	3.05
20	22	24	32.14	**2.13**	**1.64**	3.35
21	22	25	43.56	2.54	1.78	5.05
22	22	28	34.07	2.57	2.34	4.06
23	23	24	21.14	3.64	2.63	10.50
24	23	27	48.28	2.65	2.17	7.21
25	24	26	7.83	4.72	1.91	5.99
26	24	27	19.90	2.59	2.10	4.97
27	24	28	20.56	3.31	2.74	5.92
28	25	28	11.41	3.16	2.77	4.29
29	26	27	11.66	2.32	1.79	5.01
30	29	31	32.80	3.45	2.63	5.91
31	30	32	9.24	2.65	1.93	6.24
32	30	34	20.49	**2.04**	**1.69**	4.46
33	31	37	38.56	3.40	2.61	6.19
34	32	33	6.24	2.68	1.92	4.53
35	32	34	9.66	3.19	1.87	7.45
36	33	34	3.83	7.38	4.31	11.70
37	34	36	2.00	6.82	3.41	**16.09**
38	35	36	2.00	7.43	3.71	8.79
39	35	37	1.41	2.78	1.96	5.76
40	36	38	1.41	3.80	2.68	4.47
41	36	39	2.41	10.50	4.35	10.40
42	38	39	5.41	6.36	4.70	11.66

*Note:* Bolded values indicate parameters that achieved relative optimal performance for a specific metric across all corridors.

**FIGURE 4:**
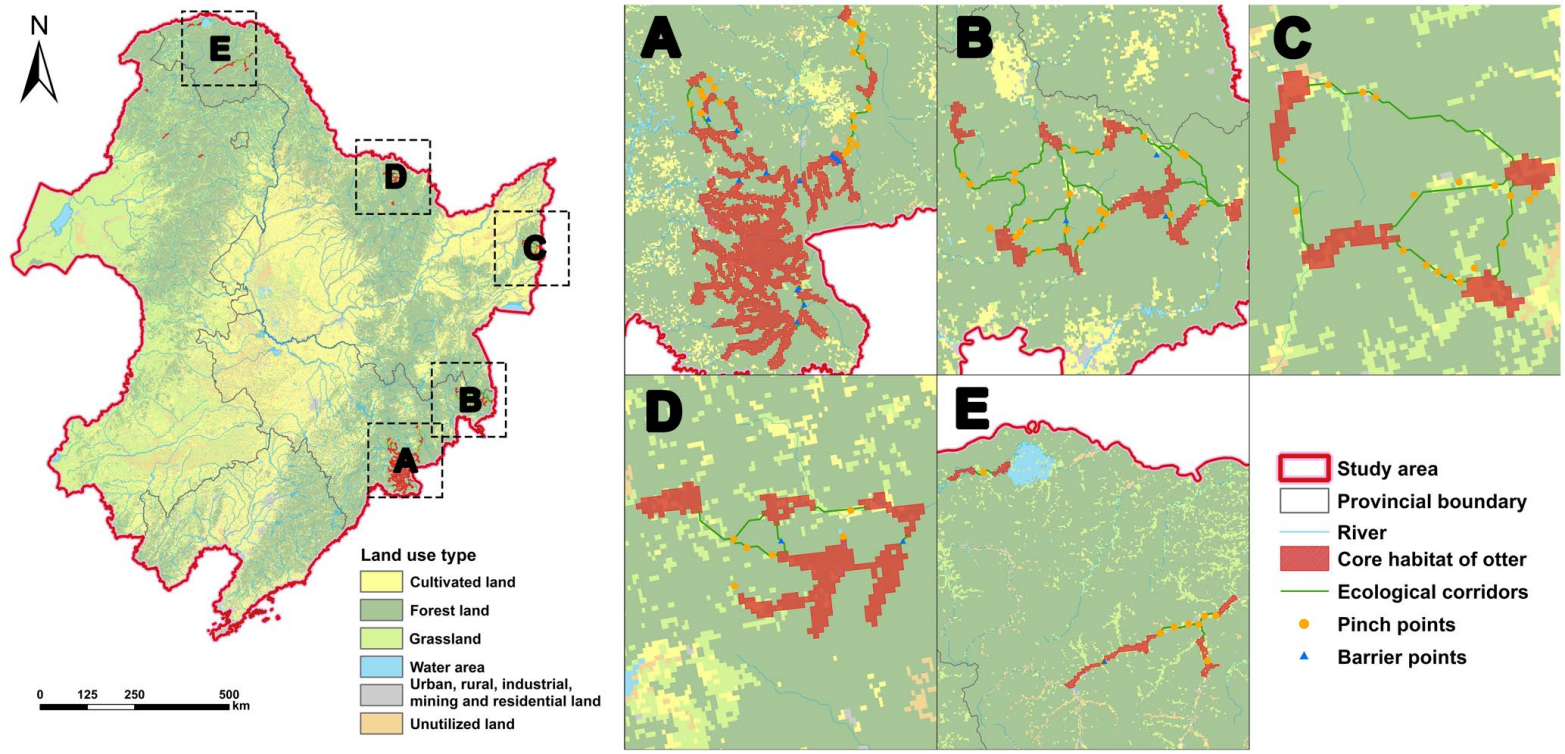
Distribution of 42 ecological corridors, 78 pinch points, and 19 barrier points. (A) The Changbai Mountains; (B) The Panling Mountains; (C) The Wanda Mountains; (D) The Wuyiling Mountains; (E) The northern Daxing'anling Mountains.

A total of 78 ecological pinch points and 19 ecological barrier points were identified in this study (Figure 4). Among these, 57 ecological pinch points and 17 ecological barrier points were located in the woodland within forest land (Table A2). The results strongly indicated that proximity to rivers is the primary environmental driver distinguishing barrier points within the corridor. While the density of forest land, the density of cultivated land, and precipitation seasonality were important factors in the overall resistance surface influencing broad‐scale movement costs, they did not significantly differ between specific barrier points and random points within the corridors themselves (Figure S1).

### 3.4 Priority Conservation Areas

**FIGURE 5:**
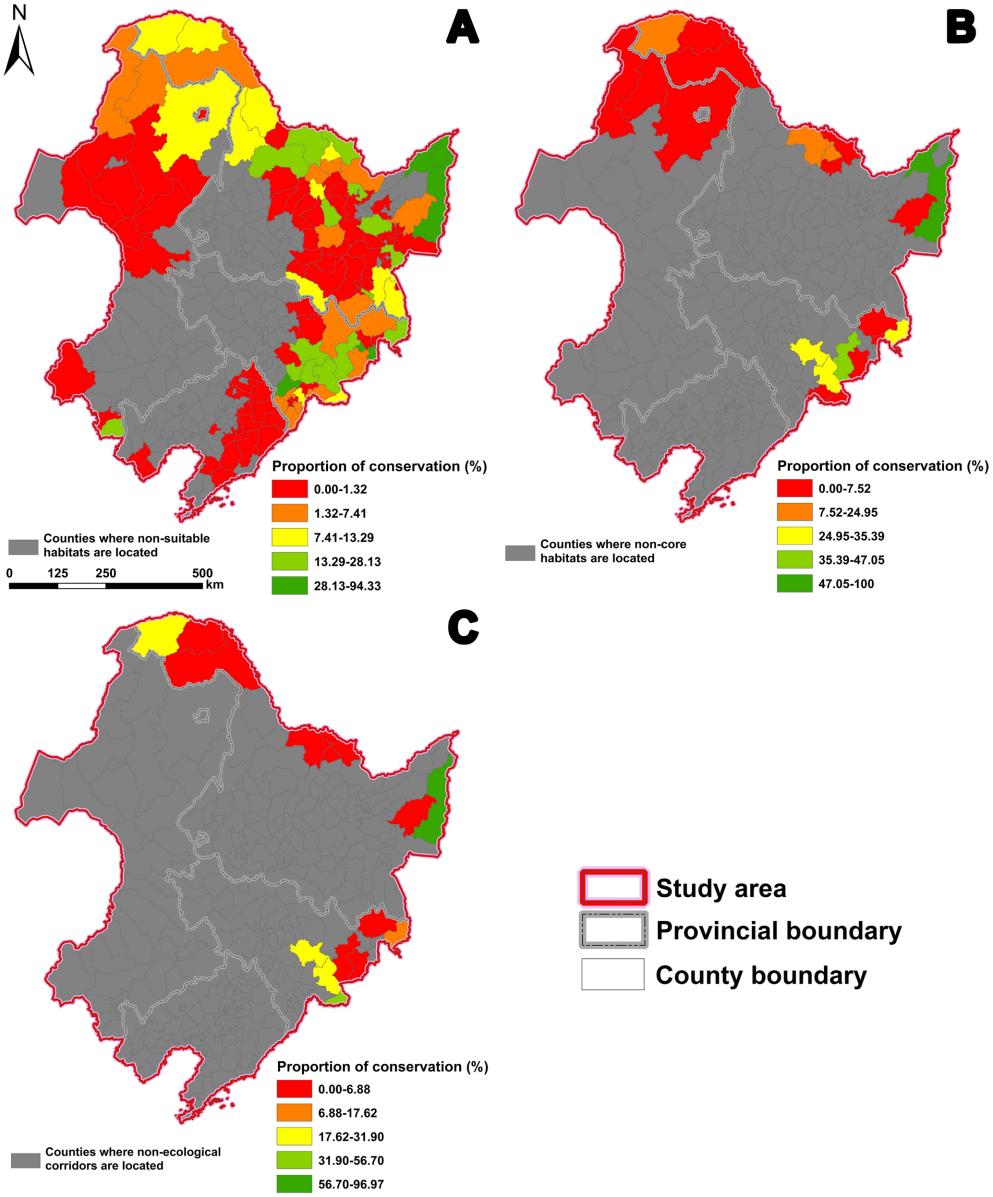
The protection proportion of suitable habitat (A), core habitat (B), and corridors (C) by each county. Using the natural breaks method (Jenks), the data were classified into five tiers, with red indicating a relatively low protection proportion and green indicating a relatively high protection proportion.

The area of suitable habitats for the Eurasian otter in the national nature reserves in the study area was 13,000.00 km^2^, and the proportion of suitable habitats under protection was 11.92%. From a county‐level perspective, Raohe County (Shuangyashan City, Heilongjiang Province) had the highest proportion of suitable habitats protected, at 94.33%. However, there were 56 county‐level areas with less than 50% protection, and 50 county‐level areas did not have national nature reserves, especially the counties around the Northeast Plain (Table A3; Figure 5A).

The area of protected core habitats was 1293.05 km^2^, accounting for 25.83% of the total area of core habitats. Among them, three county‐level areas had more than 50% of core habitats protected, including Tongjiang City (Jiamusi City, Heilongjiang Province), where 100% of the area was protected (Table A4). However, county‐level regions located in the Daxing'anling Mountains lacked protection for the core habitats of the Eurasian otter (Figure 5B).

The corridors within the national nature reserves totaled 150.16 km, accounting for 18.66% of the total corridor length. In terms of county‐level areas, Raohe County (Shuangyashan City, Heilongjiang Province), Hulin City (Jixi City, Heilongjiang Province), and Changbai Korean Autonomous County (Baishan City, Jilin Province), had higher protection ratios of 96.97%, 79.48%, and 56.70%, respectively, but there were 9 county‐level areas with protection ratios of less than 10% (Table A5), which were mainly located in the northern part of the Daxing'anling Mountain, the eastern side of the Xiaoxing'anling Mountain, the southern part of the Sanjiang Plain and the Changbai Mountain (Figure 5C).

## 4 Discussion

### 4.1 Species Distribution Model and Suitable Habitats

Compared with previous studies (Chen, Zhang, Fu, et al. 2023; Chen, Zhang, Wang, et al. 2023; Lv et al. 2018; Zhang, Chen, et al. 2022), this research not only utilized site data obtained through field surveys and literature collection but also incorporated open biodiversity data from sources such as the GBIF and iNaturalist. These additional data sources have further enriched the distribution records of the Eurasian otter in the study area. The aggregation and sharing of scientific data are conducive to promoting scientific development and innovation in research paradigms. In particular, the application of citizen science data in the field of biodiversity has seen exponential growth in recent years (Della Rocca et al. 2024). Although open biodiversity databases provide researchers with a low‐cost and multi‐scale source of species information, the varying levels of expertise among citizen scientists may affect the accuracy and reliability of the data (Della Rocca et al. 2024). Additionally, the uneven spatial distribution of data—where more developed regions tend to have richer data—and the insufficiency of species absence data can also limit the model's ability to interpret species distribution patterns (Amano et al. 2016; Della Rocca et al. 2024). Therefore, integrating data from different sources and ensuring their comprehensiveness and accuracy remain areas that require new solutions for researchers (Fletcher Jr et al. 2019; Isaac et al. 2020).

The Eurasian otter, a semi‐aquatic mammal, inhabits freshwater and riparian ecosystems and primarily preys on fish (Fu et al. 2024; Zhan et al. 2023). Based on the species' biological traits, this study incorporated bioclimatic, topographic, human disturbance, land use, and water‐related environmental variables to predict its distribution. Among these, distance from rivers emerged as the most critical factor influencing the species' distribution. Eurasian otters predominantly occupied upstream river areas with minimal human disturbance, such as the Emur River, Huma River, Jiliu River, Gan River, and Gen River in the Daxing'anling Mountain; the Tangwang River and Hulan River in the Xiaoxing'anling Mountain; and the Muling River, Suifen River, Tumen River, and Hun River in the Changbai Mountains. Due to practical constraints in the study, fine‐scale environmental data, like riparian land cover, river width and depth, flow velocity, water quality, and fish biomass were not fully obtained, despite their potential influence on otter distribution (Loy et al. 2009; Balestrieri et al. 2016; Cianfrani et al. 2010; Hong et al. 2018; Jo et al. 2017; Remonti et al. 2008). Microhabitat studies can provide targeted insights into the species' habitat preferences and utilization (Martin 1998). For instance, Eurasian otters in Poland primarily feed on smaller fish (Kloskowski et al. 2013), while those in southern Italy show a preference for chub ( *Leuciscus cephalus* ) and avoid bleak (*Alburnus* sp.) (Remonti et al. 2010). In the Central Russian Upland, access to open water and water levels during snowy and snow‐free periods are key factors affecting otter behavior (Sokolova et al. 2022). The complexity of habitat selection for species underscores the importance of exploring underlying mechanisms for biodiversity conservation.

In this study, the suitable habitats for Eurasian otters were primarily distributed along rivers in high‐altitude mountainous regions including the Daxing'anling Mountain, Xiaoxing'anling Mountain, and the Changbai Mountains, covering an area of 109,400.00 km^2^. This finding was consistent with a previous study (Zhang, Chen, et al. 2022). Approximately 70% of the suitable habitats for Eurasian otters were located in forest land, particularly in closed forests with a coverage rate of over 30%. Previous studies have shown that the occurrence rate of Eurasian otters increases with forest density and altitude (Hong and Joo 2021; Hong et al. 2018), and the species prefer to reside in river segments within forested areas (Dettori et al. 2022). The riparian vegetation provides an ideal habitat for otters, and a higher forest coverage rate generally results in better water quality (Qiu et al. 2023), thereby creating more favorable conditions for otters' survival.

Regarding human pressures, our study revealed a significant negative impact of cropland on otter presence in Northeast China, while influences from roads, dams, and human population density were less pronounced. This pattern contrasts with findings from several European and Asian regions. For instance, studies (Romanowski 2013) in central and eastern Poland reported a positive association between otter signs and bridges (indicating potential use of under‐bridge structures or tolerance of associated disturbance). In Iran, otter occurrence showed a significant positive correlation with the presence of artificial ponds (Mirzaei et al. 2009). Similarly, research in South Korea documented otters adapting to and utilizing areas near roads, residential zones, and agricultural land, suggesting a degree of tolerance to certain anthropogenic disturbances in those landscapes (Cho et al. 2009). We attribute this key difference primarily to the currently lower human population density and associated lower intensity of pervasive, multi‐faceted disturbance in the mountainous regions of Northeast China studied here. While infrastructure like roads and dams exists, their current footprint and associated human activity levels may not yet reach a threshold that strongly deters otters, unlike the significant negative impact observed from agricultural land conversion and practices (e.g., potential pollution, habitat fragmentation). This highlights a unique aspect of otter adaptation (or current lack of pressure) in our study area.

Furthermore, the Eurasian otter, whose entire existence encompasses habitat requirements, food availability, reproduction, shelter, and security, is intrinsically bound to stable, healthy freshwater ecosystems and their riparian environments. Precipitation seasonality directly undermines this fundamental stability at its core: the spatiotemporal distribution of water resources. Consequently, environments with low precipitation seasonality are generally more conducive to maintaining stable and productive aquatic ecosystems, thereby providing higher habitat suitability for otters.

Tracing the historical distribution of Eurasian otters, over the past 70 years, their distribution range in the study area has decreased by 46%, with the most significant reductions occurring in low‐altitude plains such as the Sanjiang Plain (Chen, Zhang, Fu, et al. 2023). Low‐altitude plains are more frequently disturbed by human activities, including the expansion of farmland and urban areas, as well as the associated ecological and environmental problems, like water pollution (Lu et al. 2016; Sang et al. 2023). These factors force Eurasian otters to migrate to higher‐altitude mountainous regions, particularly the Northeast China Forest Belt. More than half of the core habitats were located in Baishan city, Jilin Province, within the interior of the Changbai Mountains. Water systems are well developed within Baishan City, with numerous rivers flowing through the area. Fifty‐five large rivers, including the Yalu River, Songhua River, and Hun River, have drainage basins exceeding 100 km^2^ (Baishan Municipal People's Government 2024, accessed on 24 February 2025). The Changbai Mountains region is known for its abundant forests and food resources, providing vast and suitable habitats for otters.

### 4.2 Connectivity Analysis and Corridors

In this study, we used the minimum home range of the Eurasian otter as the threshold to identify core habitats and designate them as sources for connectivity analysis. Of the 39 core habitats, five smaller, isolated and irregularly shaped ones were not connected by corridors. These characteristics—small size, discontinuous spatial distribution, and irregular shape—can lead to further habitat fragmentation, resulting in multiple disconnected patches that are not conducive to maintaining and increasing population sizes (Penhollow and Stauffer 2000). It has been shown that connecting disconnected core habitats is more effective than protecting isolated habitat fragments (Ewers and Didham 2007).

In order to ensure the connectivity of corridors while taking into account the economic and labour costs of corridor construction, the length, width and quality of corridors are key issues in planning and design (Wang, Qin, and Önal 2022). The length of the corridor needs to take the dispersal capacity of different species into account. For species with strong dispersal capacity, most core habitats can be connected to each other to form a more complete network; while for species with limited dispersal capacity or more dispersed habitats, migration paths beyond their dispersal capacity can be identified if the dispersal capacity is not considered (Liang et al. 2023). In the study, we used the Eurasian otter movement data from previous literature and included its dispersal capacity to avoid corridors of unrealistic length. In addition to determining the dispersal capacity of species through field surveys, dispersal capacity can also be calculated through allometric growth equations based on the species' body weight and home range for terrestrial mammals (Liang et al. 2023, 2022; Santini et al. 2013). Also, corridor width is one of the key considerations. Wide corridors can provide sufficient food resources and habitats for species to guide their movement (Haddad 1999) that have greater biodiversity conservation value (Van Schalkwyk et al. 2020; Zimbres et al. 2017), while narrow corridors are more conducive to the rapid movement of species in the landscape (Kowalski et al. 2019). The width of corridors was not considered in this study due to the lack of data on the movement of species. In future studies, trajectory tracking technology (Merkle et al. 2023) can be used to obtain detailed movement data of Eurasian otters, and thus determine appropriate corridor widths.

Most corridors and pinch points were distributed along rivers in the study, which were compatible with the biological characteristics of otters. The longest corridor (Corridor 18) along the upper reaches of the Wangqing River connected two core habitats (Core 21 and 25) in Jilin Province, with relatively low mobility cost and resistance. Corridors 8, 9, 20, and 32 connected the otter's sources along the branches of the Ating River, Wuyun River, Suifen River, and Erdaoliu River, respectively. Core habitats (Core 34 and 36), connected by Corridor 37 in Baishan City, Jilin Province, were relatively close in space, with more potential dispersal paths between them.

Furthermore, we found that the distance from rivers is the main factor in the formation of barrier points. Out of the 19 barrier points, four are located within the protected areas of Yanbian Korean Autonomous Prefecture, Jilin Province. Our finding provides a clear, actionable target for management: prioritize improving connectivity in areas far from rivers within critical corridors. Strategies should focus on protecting or restoring small water features such as seasonal streams, to create “stepping stones” in these arid barrier zones, and ensuring protection of existing critical water sources near identified barriers. For output evaluation, this analysis provides a robust, statistically supported ecological rationale for the barrier points identified by our model. The significant difference specifically in distance to rivers, compared to random points within the same corridors, strongly validates the model's identification of these locations as significant impediments to movement for a water‐dependent species.

### 4.3 Conservation Applications

As otters primarily inhabit rivers, lakes, and water bodies, their management has traditionally fallen under the jurisdiction of the Ministry of Agriculture. However, since 2000, most protected areas with recorded otter presence have been managed by the State Forestry Bureau (Zhang et al. 2018). The division of management authority creates challenges for the unified conservation and management of otters and their habitats. Although protected areas in Northeast China, especially national‐level nature reserves, have played significant roles in biodiversity conservation and environmental quality improvement (Wu et al. 2022), the results in this study revealed that there were still protection gaps in the suitable habitats, core habitats, and corridors of the Eurasian otter located in the Northeast China Forest Belt. In 2017, the Ministry of Agriculture released the first batch of the Important Habitat Records of National Key Protection Aquatic Wild Animals, which included four important otter habitats: the Nuoshui River in Sichuan Province, the Taibai Xishui River in Shaanxi Province, the Danjiang Wuguan River in Shaanxi Province, and the Hei River in Shaanxi Province (Ministry of Agriculture of the People's Republic of China 2018, accessed on 24 February 2025). This initiative has brought hope for further conservation of the Eurasian otter in China. In the future, the Important Habitat Records of National Key Protection Aquatic Wild Animals can serve as an important pathway for the conservation of the otters in Northeast China and also as an effective supplement to the existing protected area network.

Conservation effectiveness is closely related to the size of protected areas and the intensity of conservation measures. For endangered species, increased protected area size generally enhances conservation outcomes. Moreover, strict conservation measures are more effective for species survival compared to general protection efforts (Timmers et al. 2022). Therefore, maintaining or enhancing conservation intensity is essential in addition to ensuring the adequate size of protected areas. The Northeast China Forest Belt is a vital base for forest and biological resources and an integral part of the national “Two Screens, Three Belts” ecological framework (Liao et al. 2022). However, due to human development activities, ecological land in this region has significantly decreased, while non‐ecological land has increased (Jia and Hu 2024). For instance, landscape ecological risks have risen in the Daxing'anling Mountains and the Xiaoxing'anling Mountains (Sui et al. 2024). Habitat degradation caused by land use changes is one of the significant threats to species. Research has predicted that land use changes alone could lead to the endangerment of approximately 1700 species globally (Powers and Jetz 2019). Furthermore, some habitats and ecological corridors are not included in protected areas, making them more susceptible to human disturbances. For example, the Mesoamerican Biological Corridor (CBM‐M), Kwakuchinja Wildlife Corridor (KWC), and Wami Mbiki‐Saadani (WMS) wildlife corridor are facing risks of reduced ecological connectivity due to land use changes (Díaz‐Gallegos et al. 2008; Martin et al. 2019; Ntukey et al. 2022), which should alert researchers and policymakers. Additionally, as a semi‐aquatic mammal, the Eurasian otter relies heavily on water and aquatic food resources for survival. Water pollution caused by factors such as pesticide misuse directly leads to the bioaccumulation of pollutants in otters (Mucci et al. 2010), which are at the top of the freshwater ecosystem food chain. Moreover, reduced biomass of aquatic animals due to water pollution further exacerbates the decline in otter populations (Piao et al. 2011).

The natural dispersion of species is not solely dependent on corridors but also heavily influenced by the matrix (Baum et al. 2004). The Drift Fence hypothesis posits that corridors may only be partially utilized for species dispersal, facilitating movement from one suitable habitat through the corridor and into another suitable habitat (Fried et al. 2005). Corridors of lower quality are more suitable for long‐distance migration, while higher‐quality corridors encourage wandering, which aids in finding more food sources but reduces dispersal efficiency (Delattre et al. 2018). There is an inherent contradiction in building high‐quality corridors while also aiming to increase connectivity. Moreover, corridor permeability is constrained by the matrix (Vergara et al. 2013). If a corridor's habitat quality is subpar, it diminishes its contrast with the surrounding environment, thereby increasing the likelihood of species migrating out of the corridor and reducing corridor connectivity efficiency (Bertoncelj and Dolman 2013; Delattre et al. 2013). Models predict that corridors are most effective when the surrounding environment is of medium quality (Vergara 2011). These observations highlight the interactive relationship between corridors and matrix, underscoring the importance of integrated management of corridors and surroundings (Vos et al. 2002). For the otter, water bodies and riverbanks serve as primary migration pathways.

Moving forward, it is crucial to impose strict control over human disturbances within the Northeast China Forest Belt and to implement comprehensive protection strategies for forest and freshwater ecosystems. Efforts should also focus on restoring and managing corridors and the surrounding vegetation to enhance connectivity (Castellón and Sieving 2006), thereby establishing a robust foundation for biodiversity conservation.

## Author Contributions

**Qingyi Wang:** conceptualization (equal), data curation (equal), formal analysis (equal), writing – original draft (equal), writing – review and editing (equal). **Aihua Fu:** conceptualization (equal), data curation (equal), formal analysis (equal), writing – review and editing (equal). **Wendi Yang:** data curation (equal). **Minhao Chen:** data curation (equal), investigation (equal). **Chao Zhang:** data curation (equal), investigation (equal). **Xiaofeng Luan:** conceptualization (equal), project administration (equal), supervision (equal), writing – review and editing (equal).

## Conflicts of Interest

The authors declare no conflicts of interest.

## Supporting information


**Data S1:** ece372429‐sup‐0001‐supinfo.docx.

## Data Availability

All the required data is uploaded as Supporting Information.

## Appendix 1

**TABLE A1:** Environmental variable information.

Variable group	Variables	Resolution	Time scale	Data source
Bioclimate	Annual precipitation amount (bio12)	1 km	1981–2010	Climatologies at High Resolution for the Earth's Land Surface Areas (CHELSA), https://chelsa‐climate.org/
	Precipitation amount of the wettest month (bio13)			
	Precipitation amount of the driest month (bio14)			
	Precipitation seasonality (bio15)			
	Mean monthly precipitation amount of the wettest quarter (bio16)			
	Mean monthly precipitation amount of the driest quarter (bio17)			
	Mean monthly precipitation amount of the warmest quarter (bio18)			
	Mean monthly precipitation amount of the coldest quarter (bio19)			
Topography	Elevation	90 m	—	Geospatial Data Cloud, https://www.gscloud.cn/
	Slope			
	Aspect			
	Ruggedness			
Human Impact	Population density	1 km	2022	LandScan, https://landscan.ornl.gov/
	Distance from railways	—	2019	National Catalog Service for Geographic Information (NCSGI), https://www.webmap.cn/
	Distance from roads			
	Distance from residential areas			
	Distance from dams	—	2016	Global Dam Watch, http://www.globaldamwatch.org
Land use type	Density of cultivated land	1 km	2020	Resources and Environment Science and Data Center (RESDC), https://www.resdc.cn/
	Density of forest land			
Variables related to water area	Distance from water area	—	2019	NCSGI
	Distance from rivers			

*Note:* Water Area: All polygon features (lakes, reservoirs, wide rivers); Rivers: All line features (including streams and narrow rivers). Small ponds below the minimum mapping unit of NCSGI data are likely excluded, potentially omitting critical otter habitats.

**TABLE A2:** Proportions or numbers of suitable habitats, core habitats, corridors, and key points in different land use types.

Land‐use type (Land‐use code)		Proportion of suitable habitat (%)	Proportion of core habitat (%)	Proportion of corridors (%)	Number of pinch points	Number of barrier points
Cultivated land (1)	Paddy field (11)	0.31	0.03	0.09	0	0
	Dry field (12)	4.98	2.34	5.82	3	0
Forest land (2)	Woodland (21)	58.37	78.79	72.46	57	17
	Shrubbery (22)	6.87	3.34	2.40	0	0
	Open forest land (23)	3.49	1.20	0.09	0	0
	Other woodland (24)	0.68	2.27	0.18	0	1
Grassland (3)	High coverage grassland (31)	17.80	5.75	11.09	9	1
	Medium coverage grassland (32)	1.66	0.43	0.55	1	0
	Low coverage grassland (33)	0.04	0.00	0.00	0	0
Water area (4)	River canal (41)	0.76	0.71	0.46	1	0
	Lake (42)	0.06	0.00	0.00	0	0
	Reservoir pond (43)	0.27	0.21	0.00	0	0
	Bottomland (44)	0.23	0.05	0.09	0	0
Urban, rural, industrial, mining and residential land (5)	Urban land (51)	0.24	0.45	0.28	0	0
	Rural settlement (52)	0.49	0.33	0.92	1	0
	Other construction land (53)	0.07	0.23	0.00	0	0
Unutilized land (6)	Wetland (61)	3.64	3.44	5.55	6	0
	Bare land (62)	0.03	0.00	0.00	0	0
	Bare gravelly land (63)	0.02	0.01	0.00	0	0

**TABLE A3:** The distribution of suitable habitats and conservation status across different administrative regions.

Province	Prefecture	County	Area of suitable habitats (km^2^)	Proportion of suitable habitats (%)	Proportion of suitable habitats under protection (%)
Heilongjiang Province			58,632.21	53.58	14.13
	Daxing'anling Mountains area		20,205.60	18.46	8.45
		Huma County	8579.00	7.84	6.89
		Jiagedaqi District	36.93	0.03	0.00
		Mohe City	6386.07	5.84	9.64
		Tahe County	5203.59	4.76	9.62
	Harbin City		2637.08	2.41	3.83
		Acheng District	13.40	0.01	0.00
		Bin County	1.73	0.00	0.00
		Fangzheng County	268.31	0.25	0.00
		Mulan County	79.50	0.07	0.00
		Shangzhi City	926.46	0.85	0.00
		Tonghe City	650.15	0.59	6.51
		Wuchang City	500.40	0.46	11.71
		Yanshou County	101.33	0.09	0.00
		Yilan County	95.78	0.09	0.01
	Hegang City		1926.36	1.76	8.39
		Dongshan District	240.45	0.22	24.94
		Luobei County	1682.30	1.54	6.05
		Xing'an District	3.61	0.00	0.00
	Heihe City		9095.53	8.31	18.99
		Aihui District	2861.72	2.62	9.01
		Beian City	173.38	0.16	0.02
		Nenjiang City	754.12	0.69	11.29
		Sunwu County	184.65	0.17	0.25
		Wudalianchi City	2069.85	1.89	27.88
		Xunke County	3051.81	2.79	26.42
	Jixi City		1482.87	1.36	52.40
		Chengzihe District	0.40	0.00	0.00
		Didao District	1.83	0.00	0.00
		Hengshan District	37.85	0.03	0.00
		Hulin City	1141.84	1.04	61.66
		Jidong County	261.86	0.24	27.87
		Lishu District	23.65	0.02	0.00
		Mishan City	15.45	0.01	0.00
	Jiamusi City		1614.58	1.48	47.56
		Fuyuan City	569.61	0.52	53.06
		Huanan County	390.27	0.36	27.57
		Tangyuan County	160.75	0.15	0.00
		Tongjiang City	493.94	0.45	72.49
	Mudanjiang City		7791.19	7.12	7.06
		Aimin District	4.27	0.00	0.00
		Dong'an District	167.32	0.15	22.65
		Dongning City	2438.59	2.23	12.29
		Hailin City	1919.56	1.75	0.00
		Linkou County	325.77	0.30	0.00
		Muling City	1359.11	1.24	11.31
		Ning'an City	1372.30	1.25	4.27
		Suifenhe City	150.51	0.14	0.00
		Xian District	0.45	0.00	0.00
		Yangming District	53.32	0.05	0.03
	Qitaihe City		42.61	0.04	0.00
		Boli County	19.50	0.02	0.00
		Qiezihe District	0.89	0.00	0.00
		Xinxing District	22.22	0.02	0.00
	Shuangyashan City		2260.13	2.07	61.7
		Baoqing County	707.22	0.65	4.79
		Jixian County	9.89	0.01	0.00
		Lingdong District	100.50	0.09	0.00
		Raohe County	1442.52	1.32	94.33
	Suihua City		781.98	0.71	0.00
		Hailun City	18.78	0.02	0.00
		Qing'an County	413.78	0.38	0.00
		Suileng County	349.42	0.32	0.00
	Yichun City		10,794.28	9.86	10.16
		Daqingshan County	1145.62	1.05	19.07
		Fenglin County	1273.98	1.16	4.52
		Jiayin County	2560.97	2.34	21.86
		Jinlin District	794.15	0.73	0.01
		Nancha County	848.6	0.78	0.00
		Tangwang County	1119.34	1.02	13.29
		Tieli City	519.83	0.48	0.00
		Wucui District	776.24	0.71	9.10
		Yimei District	956.81	0.87	0.27
		Youhao District	798.73	0.73	4.80
Jilin Province			23,524.97	21.5	14.61
	Baishan City		8173.77	7.47	17.53
		Fusong County	3822.91	3.49	28.13
		Hunjiang District	275.54	0.25	12.11
		Jiangyuan District	525.48	0.48	0.01
		Jingyu County	884.33	0.81	20.94
		Linjiang City	1414.92	1.29	2.64
		Changbai Korean Autonomous County	1250.58	1.14	8.13
	Jilin City		1570.72	1.44	13.77
		Fengman District	40.17	0.04	0.70
		Huadian City	1022.93	0.93	20.79
		Jiaohe City	343.2	0.31	0.95
		Panshi City	0.63	0.00	0.00
		Shulan City	163.78	0.15	0.00
	Tonghua City		1288.79	1.18	16.44
		Dongchang District	18.26	0.02	0.00
		Erdaojiang District	51.43	0.05	0.00
		Huinan County	194.80	0.18	26.21
		Jian City	475.71	0.43	5.81
		Liuhe County	235.61	0.22	48.08
		Tonghua County	312.99	0.29	6.37
	Yanbian Korean Autonomous Prefecture		12,491.68	11.42	12.62
		Antu County	2986.10	2.73	21.54
		Dunhua City	2270.10	2.07	7.41
		Helong City	1405.08	1.28	2.60
		Hunchun City	1895.24	1.73	24.21
		Longjing City	215.29	0.20	70.98
		Tumen City	275.68	0.25	0.00
		Wangqing County	3213.51	2.94	3.64
		Yanji City	230.69	0.21	0.00
Liaoning Province			1379.72	1.26	0.25
	Anshan City		0.85	0.00	0.00
		Xiuyan Manchu Autonomous County	0.85	0.00	0.00
	Benxi City		451.19	0.41	0.15
		Benxi Manchu Autonomous County	184.46	0.17	0.00
		Huanren Manchu Autonomous County	239.22	0.22	0.28
		Mingshan District	1.29	0.00	0.00
		Nanfen District	26.22	0.02	0.00
	Dalian City		0.66	0.00	0.00
		Zhuanghe City	0.66	0.00	0.00
	Dandong City		389.49	0.36	0.17
		Fengcheng City	19.46	0.02	0.00
		Kuandian Manchu Autonomous County	370.03	0.34	0.18
	Fushun City		484.95	0.44	0.44
		Fushun County	90.14	0.08	0.00
		Qingyuan Manchu Autonomous County	64.01	0.06	0.00
		Xinbin Manchu Autonomous County	330.80	0.30	0.64
	Huludao City		7.21	0.01	0.00
		Jianchang County	4.38	0.00	0.00
		Suizhong County	2.83	0.00	0.00
	Liaoyang City		16.88	0.02	0.00
		Liaoyang County	16.88	0.02	0.00
	Tieling City		4.40	0.00	0.00
		Kaiyuan City	0.64	0.00	0.00
		Tieling County	3.13	0.00	0.00
		Xifeng County	0.63	0.00	0.00
	Yingkou City		24.07	0.02	0.00
		Gaizhou City	24.07	0.02	0.00
Inner Mongolia Autonomous Region			25,890.35	23.66	5.09
	Chifeng City		43.9	0.04	12.71
		Kalaqin Banner	14.16	0.01	0.00
		Keshketeng Banner	1.27	0.00	0.00
		Ningcheng County	28.47	0.03	19.59
	Hulun Buir City		24,674.09	22.55	5.32
		Arong Banner	28.8	0.03	0.00
		Chen Barhu Banner	149.35	0.14	0.00
		Erguna City	5733.56	5.24	4.38
		Oroqen Autonomous Banner	7650.87	6.99	11.22
		Ewenk Autonomous Banner	1341.73	1.23	1.32
		Genhe City	4874.49	4.45	3.79
		New Barag East Banner	282.23	0.26	0.00
		Yakeshi City	4228.03	3.86	0.00
		Zhalantun City	385.04	0.35	0.00
	Xing'an League		1172.37	1.07	0.00
		Alshan City	1136.91	1.04	0.00
		Horqin Right Front Banner	35.45	0.03	0.00
Total			109,427.25	100.00	11.92

*Note:* Proportion of suitable habitats refers to the ratio of the area of suitable habitats in the region to the total area of suitable habitats. Proportion of suitable habitats under protection is the ratio of the area of suitable habitats within protected areas to the area of suitable habitats in the region.

**TABLE A4:** The distribution of core habitats and conservation status across different administrative regions.

Province	Prefecture	County	Area of core habitats (km^2^)	Proportion of core habitats (%)	Proportion of core habitats under protection (%)
Heilongjiang Province			879.00	17.56	21.36
	Daxing'anling Mountains area		372.00	7.43	5.63
		Huma County	199.07	3.98	0.00
		Mohe City	84.00	1.68	24.95
		Tahe County	88.93	1.78	0.00
	Heihe City		161.78	3.23	14.67
		Xunke County	161.78	3.23	14.67
	Jixi City		95.59	1.91	83.01
		Hulin City	95.59	1.91	83.01
	Jiamusi City		29.00	0.58	100.00
		Tongjiang City	29.00	0.58	100.00
	Shuangyashan City		57.41	1.15	26.19
		Baoqing County	38.51	0.77	0.21
		Raohe County	18.90	0.38	79.14
	Yichun City		163.22	3.26	12.07
		Fenglin County	30.00	0.60	0.00
		Jiayin County	21.70	0.43	5.00
		Tangwang County	111.52	2.23	16.69
Jilin Province			3998.00	79.86	27.65
	Baishan City		2940.38	58.74	25.29
		Fusong County	2024.83	40.45	35.39
		Linjiang City	431.92	8.63	0.00
		Changbai Korean Autonomous County	483.63	9.66	5.63
	Jilin City		52.35	1.05	34.11
		Huadian City	52.35	1.05	34.11
	Yanbian Korean Autonomous Prefecture		1005.27	20.08	34.18
		Antu County	545.64	10.9	47.05
		Helong City	26.63	0.53	0.00
		Hunchun City	221.47	4.42	32.47
		Tumen City	11.91	0.24	0.00
		Wangqing County	199.62	3.99	7.52
Inner Mongolia Autonomous Region			129.00	2.58	0.00
	Hulunbuir City		129.00	2.58	0.00
		Erguna City	29.00	0.58	0.00
		Oroqen Autonomous Banner	53.00	1.06	0.00
		Genhe City	47.00	0.94	0.00
Total			5006.00	100.00	25.83

*Note:* Proportion of core habitats refers to the ratio of the area of core habitats in the region to the total area of core habitats. Proportion of core habitats under protection is the ratio of the area of core habitats within protected areas to the area of core habitats in the region.

**TABLE A5:** The distribution of corridors and conservation status across different administrative regions.

Province	Prefecture	County	Length of corridors (km)	Proportion of corridors (%)	Proportion of corridors under protection (%)
Heilongjiang Province			293.18	36.43	30.98
	Daxing'anling Mountains area		126.57	15.73	2.76
		Huma County	23.31	2.90	0.00
		Mohe City	12.49	1.55	28.03
		Tahe County	90.78	11.28	0.00
	Heihe City		46.46	5.77	0.00
		Xunke County	46.46	5.77	0.00
	Jixi City		61.20	7.60	79.48
		Hulin City	61.20	7.60	79.48
	Shuangyashan City		49.13	6.10	78.77
		Baoqing County	9.22	1.15	0.00
		Raohe County	39.91	4.96	96.97
	Yichun City		9.82	1.22	0.00
		Jiayin County	4.53	0.56	0.00
		Tangwang County	5.30	0.66	0.00
Jilin Province			511.59	63.57	11.6
	Baishan City		30.97	3.85	38.17
		Fusong County	23.14	2.88	31.9
		Changbai Korean Autonomous County	7.83	0.97	56.7
	Jilin City		29.73	3.69	27.52
		Huadian City	29.73	3.69	27.52
	Yanbian Korean Autonomous Prefecture		450.89	56.03	8.72
		Antu County	52.42	6.51	3.82
		Helong City	22.35	2.78	0.00
		Hunchun City	106.72	13.26	17.62
		Wangqing County	269.40	33.48	6.88
Total			804.77	100.00	18.66

*Note:* Proportion of corridors refers to the ratio of the length of corridors in the region to the total length of corridors. Proportion of corridors under protection is the ratio of the length of corridors within protected areas to the length of corridors in the region.
