# Supplementary material for: Identification of Ecological Corridors for Semi‐Aquatic Vertebrates: A Case of the Eurasian Otter in Northeast China
Source: Ecol Evol. 2025 Nov 2;15(11):e72429. doi: 10.1002/ece3.72429 (PMC12580232; doi:10.1002/ece3.72429)
Supplement: Supplementary file 1 — Data S1: ece372429‐sup‐0001‐supinfo.docx. [file ECE3-15-e72429-s001.docx]

**Table S1 Occurrence points of the Eurasian otter**

| **Number** | **Longitude** | **Latitude** | **Data source** | **Coordinate uncertainty** |
| --- | --- | --- | --- | --- |
| 1 | 131.20 | 45.07 | Literature and reports (Zhang et al, 2018) | <100m |
| 2 | 131.06 | 43.41 | Literature and reports (Zhang et al, 2018) | <100m |
| 3 | 130.69 | 42.97 | Literature and reports (Zhang et al, 2018) | <50m |
| 4 | 128.01 | 42.36 | Literature and reports (Zhang et al, 2018) | <50m |
| 5 | 121.68 | 50.87 | Literature and reports (Zhang et al, 2018) | <50m |
| 6 | 128.97 | 47.10 | Field surveys | <500m |
| 7 | 129.05 | 47.81 | Field surveys | <500m |
| 8 | 129.26 | 47.74 | Field surveys | <500m |
| 9 | 129.45 | 47.90 | Field surveys | <500m |
| 10 | 128.40 | 48.48 | Field surveys | <500m |
| 11 | 128.63 | 48.78 | Field surveys | <500m |
| 12 | 128.98 | 48.75 | Field surveys | <500m |
| 13 | 129.41 | 48.59 | Field surveys | <500m |
| 14 | 129.70 | 48.50 | Field surveys | <500m |
| 15 | 129.38 | 49.37 | Field surveys | <500m |
| 16 | 129.64 | 49.09 | Field surveys | <500m |
| 17 | 129.88 | 48.89 | Field surveys | <500m |
| 18 | 129.75 | 48.93 | Field surveys | <500m |
| 19 | 126.92 | 50.35 | Field surveys | <500m |
| 20 | 127.23 | 49.87 | Field surveys | <500m |
| 21 | 127.02 | 49.55 | Field surveys | <500m |
| 22 | 126.62 | 48.90 | Field surveys | <500m |
| 23 | 127.21 | 48.44 | Field surveys | <500m |
| 24 | 127.94 | 49.46 | Field surveys | <500m |
| 25 | 129.05 | 46.73 | Field surveys | <500m |
| 26 | 129.16 | 46.83 | Field surveys | <500m |
| 27 | 127.16 | 42.59 | Field surveys | <500m |
| 28 | 134.53 | 48.02 | Field surveys | <500m |
| 29 | 134.45 | 48.37 | Field surveys | <500m |
| 30 | 133.83 | 48.24 | Field surveys | <500m |
| 31 | 134.61 | 47.55 | Field surveys | <500m |
| 32 | 133.08 | 48.06 | Field surveys | <500m |
| 33 | 132.21 | 45.95 | Field surveys | <500m |
| 34 | 131.22 | 44.21 | Field surveys | <500m |
| 35 | 130.02 | 43.59 | Field surveys | <500m |
| 36 | 130.44 | 43.52 | Field surveys | <500m |
| 37 | 126.24 | 41.31 | Field surveys | <500m |
| 38 | 127.98 | 42.28 | Field surveys | <500m |
| 39 | 126.38 | 42.30 | Field surveys | <500m |
| 40 | 128.72 | 44.12 | Field surveys | <500m |
| 41 | 133.83 | 46.28 | Field surveys | <500m |
| 42 | 126.11 | 42.10 | Field surveys | <500m |
| 43 | 128.01 | 44.18 | Field surveys | <500m |
| 44 | 131.02 | 43.70 | Field surveys | <500m |
| 45 | 126.56 | 41.76 | Field surveys | <500m |
| 46 | 128.09 | 43.90 | Field surveys | <500m |
| 47 | 130.41 | 43.18 | Field surveys | <500m |
| 48 | 123.29 | 51.58 | Field surveys | <500m |
| 49 | 122.74 | 51.51 | Field surveys | <500m |
| 50 | 121.32 | 50.68 | Field surveys | <500m |
| 51 | 122.62 | 50.24 | Field surveys | <500m |
| 52 | 124.34 | 50.62 | Field surveys | <500m |
| 53 | 124.30 | 50.86 | Field surveys | <500m |
| 54 | 124.00 | 50.85 | Field surveys | <500m |
| 55 | 128.09 | 42.34 | Field surveys | <500m |
| 56 | 126.88 | 49.59 | Field surveys | <500m |
| 57 | 126.32 | 50.27 | Field surveys | <500m |
| 58 | 121.24 | 47.69 | Field surveys | <500m |
| 59 | 120.32 | 47.29 | Field surveys | <500m |
| 60 | 120.66 | 47.48 | Field surveys | <500m |
| 61 | 120.59 | 48.45 | Field surveys | <500m |
| 62 | 120.81 | 49.24 | Field surveys | <500m |
| 63 | 122.89 | 51.20 | Field surveys | <500m |
| 64 | 122.98 | 50.93 | Field surveys | <500m |
| 65 | 123.08 | 50.73 | Field surveys | <500m |
| 66 | 123.62 | 50.17 | Field surveys | <500m |
| 67 | 125.30 | 51.79 | Field surveys | <500m |
| 68 | 124.12 | 51.42 | Field surveys | <500m |
| 69 | 123.40 | 51.64 | Field surveys | <500m |
| 70 | 125.28 | 51.13 | Field surveys | <500m |
| 71 | 124.22 | 51.61 | Field surveys | <500m |
| 72 | 121.46 | 53.20 | Field surveys | <500m |
| 73 | 121.40 | 53.01 | Field surveys | <500m |
| 74 | 121.25 | 52.45 | Field surveys | <500m |
| 75 | 121.18 | 52.18 | Field surveys | <500m |
| 76 | 120.82 | 51.99 | Field surveys | <500m |
| 77 | 121.00 | 51.81 | Field surveys | <500m |
| 78 | 120.55 | 50.72 | Field surveys | <500m |
| 79 | 120.65 | 50.76 | Field surveys | <500m |
| 80 | 120.77 | 51.35 | Field surveys | <500m |
| 81 | 120.59 | 51.38 | Field surveys | <500m |
| 82 | 120.48 | 47.27 | Field surveys | <500m |
| 83 | 121.16 | 47.80 | Field surveys | <500m |
| 84 | 120.76 | 48.33 | Field surveys | <500m |
| 85 | 122.55 | 53.22 | Field surveys | <500m |
| 86 | 122.95 | 53.33 | Field surveys | <500m |
| 87 | 122.05 | 52.17 | Field surveys | <500m |
| 88 | 120.75 | 52.62 | Field surveys | <500m |
| 89 | 121.05 | 52.70 | Field surveys | <500m |
| 90 | 121.21 | 52.23 | Field surveys | <500m |
| 91 | 121.46 | 51.20 | Field surveys | <500m |
| 92 | 122.65 | 51.47 | Field surveys | <500m |
| 93 | 121.77 | 51.56 | Field surveys | <500m |
| 94 | 121.48 | 51.36 | Field surveys | <500m |
| 95 | 121.38 | 51.29 | Field surveys | <500m |
| 96 | 121.30 | 51.37 | Field surveys | <500m |
| 97 | 121.61 | 50.83 | Field surveys | <500m |
| 98 | 122.73 | 49.94 | Field surveys | <500m |
| 99 | 122.73 | 49.98 | Field surveys | <500m |
| 100 | 122.51 | 50.39 | Field surveys | <500m |
| 101 | 122.63 | 50.28 | Field surveys | <500m |
| 102 | 122.59 | 50.65 | Field surveys | <500m |
| 103 | 122.69 | 50.66 | Field surveys | <500m |
| 104 | 122.82 | 50.64 | Field surveys | <500m |
| 105 | 123.15 | 49.47 | Field surveys | <500m |
| 106 | 120.93 | 51.89 | Field surveys | <500m |
| 107 | 120.24 | 47.47 | Field surveys | <500m |
| 108 | 121.25 | 48.11 | Field surveys | <500m |
| 109 | 122.90 | 51.11 | Field surveys | <500m |
| 110 | 122.99 | 51.13 | Field surveys | <500m |
| 111 | 122.96 | 50.88 | Field surveys | <500m |
| 112 | 122.96 | 50.84 | Field surveys | <500m |
| 113 | 123.12 | 50.65 | Field surveys | <500m |
| 114 | 122.84 | 51.27 | Field surveys | <500m |
| 115 | 123.02 | 50.79 | Field surveys | <500m |
| 116 | 122.51 | 50.13 | Field surveys | <500m |
| 117 | 120.90 | 49.20 | Field surveys | <500m |
| 118 | 121.22 | 47.93 | Field surveys | <500m |
| 119 | 120.62 | 48.64 | Field surveys | <500m |
| 120 | 120.63 | 48.41 | Field surveys | <500m |
| 121 | 125.14 | 51.29 | Field surveys | <500m |
| 122 | 125.30 | 51.38 | Field surveys | <500m |
| 123 | 122.88 | 51.17 | Field surveys | <500m |
| 124 | 124.40 | 51.61 | Field surveys | <500m |
| 125 | 123.56 | 52.07 | Field surveys | <500m |
| 126 | 123.61 | 51.99 | Field surveys | <500m |
| 127 | 122.91 | 51.86 | Field surveys | <500m |
| 128 | 123.01 | 51.77 | Field surveys | <500m |
| 129 | 123.21 | 51.59 | Field surveys | <500m |
| 130 | 123.75 | 52.18 | Field surveys | <500m |
| 131 | 123.48 | 52.08 | Field surveys | <500m |
| 132 | 124.08 | 52.85 | Field surveys | <500m |
| 133 | 124.92 | 52.38 | Field surveys | <500m |
| 134 | 124.11 | 52.96 | Field surveys | <500m |
| 135 | 125.38 | 52.48 | Field surveys | <500m |
| 136 | 125.63 | 52.89 | Field surveys | <500m |
| 137 | 125.63 | 52.04 | Field surveys | <500m |
| 138 | 123.97 | 52.80 | Field surveys | <500m |
| 139 | 123.86 | 52.68 | Field surveys | <500m |
| 140 | 125.18 | 53.18 | Field surveys | <500m |
| 141 | 123.23 | 53.33 | Field surveys | <500m |
| 142 | 122.75 | 52.92 | Field surveys | <500m |
| 143 | 121.86 | 51.56 | Field surveys | <500m |
| 144 | 125.41 | 51.19 | Field surveys | <500m |
| 145 | 124.99 | 40.57 | Field surveys | <500m |
| 146 | 126.18 | 42.13 | Field surveys | <500m |
| 147 | 126.11 | 42.26 | Field surveys | <500m |
| 148 | 126.37 | 42.19 | Field surveys | <500m |
| 149 | 126.48 | 42.23 | Field surveys | <500m |
| 150 | 127.61 | 42.62 | Field surveys | <500m |
| 151 | 127.52 | 42.56 | Field surveys | <500m |
| 152 | 126.64 | 42.30 | Field surveys | <500m |
| 153 | 126.90 | 42.26 | Field surveys | <500m |
| 154 | 127.35 | 42.64 | Field surveys | <500m |
| 155 | 127.35 | 42.59 | Field surveys | <500m |
| 156 | 127.48 | 42.65 | Field surveys | <500m |
| 157 | 127.71 | 41.97 | Field surveys | <500m |
| 158 | 127.71 | 41.94 | Field surveys | <500m |
| 159 | 127.62 | 41.99 | Field surveys | <500m |
| 160 | 127.19 | 41.86 | Field surveys | <500m |
| 161 | 127.75 | 41.78 | Field surveys | <500m |
| 162 | 127.95 | 41.53 | Field surveys | <500m |
| 163 | 128.17 | 41.49 | Field surveys | <500m |
| 164 | 128.02 | 41.84 | Field surveys | <500m |
| 165 | 127.85 | 41.90 | Field surveys | <500m |
| 166 | 127.78 | 41.85 | Field surveys | <500m |
| 167 | 127.89 | 41.97 | Field surveys | <500m |
| 168 | 127.98 | 42.31 | Field surveys | <500m |
| 169 | 127.84 | 41.85 | Field surveys | <500m |
| 170 | 128.18 | 42.28 | Field surveys | <500m |
| 171 | 128.04 | 42.41 | Field surveys | <500m |
| 172 | 127.98 | 42.25 | Field surveys | <500m |
| 173 | 128.11 | 42.46 | Field surveys | <500m |
| 174 | 128.11 | 42.43 | Field surveys | <500m |
| 175 | 128.09 | 42.25 | Field surveys | <500m |
| 176 | 128.12 | 42.16 | Field surveys | <500m |
| 177 | 127.55 | 42.16 | Field surveys | <500m |
| 178 | 127.74 | 42.07 | Field surveys | <500m |
| 179 | 127.82 | 42.03 | Field surveys | <500m |
| 180 | 127.80 | 41.97 | Field surveys | <500m |
| 181 | 127.72 | 42.24 | Field surveys | <500m |
| 182 | 127.84 | 42.21 | Field surveys | <500m |
| 183 | 127.91 | 42.16 | Field surveys | <500m |
| 184 | 128.12 | 42.12 | Field surveys | <500m |
| 185 | 128.17 | 42.23 | Field surveys | <500m |
| 186 | 128.20 | 42.59 | Field surveys | <500m |
| 187 | 128.06 | 42.66 | Field surveys | <500m |
| 188 | 128.19 | 42.71 | Field surveys | <500m |
| 189 | 128.30 | 42.84 | Field surveys | <500m |
| 190 | 128.40 | 42.80 | Field surveys | <500m |
| 191 | 128.38 | 42.72 | Field surveys | <500m |
| 192 | 128.50 | 42.72 | Field surveys | <500m |
| 193 | 131.10 | 43.34 | Field surveys | <500m |
| 194 | 131.15 | 43.25 | Field surveys | <500m |
| 195 | 131.17 | 43.33 | Field surveys | <500m |
| 196 | 129.87 | 43.94 | Field surveys | <500m |
| 197 | 128.53 | 43.22 | Field surveys | <500m |
| 198 | 130.87 | 43.70 | Field surveys | <500m |
| 199 | 130.48 | 43.36 | Field surveys | <500m |
| 200 | 130.71 | 43.29 | Field surveys | <500m |
| 201 | 128.63 | 44.24 | Field surveys | <500m |
| 202 | 128.54 | 44.16 | Field surveys | <500m |
| 203 | 130.12 | 43.95 | Field surveys | <500m |
| 204 | 130.08 | 43.87 | Field surveys | <500m |
| 205 | 130.41 | 44.15 | Field surveys | <500m |
| 206 | 129.94 | 44.27 | Field surveys | <500m |
| 207 | 130.86 | 43.51 | Field surveys | <500m |
| 208 | 131.09 | 43.71 | Field surveys | <500m |
| 209 | 130.97 | 44.64 | Field surveys | <500m |
| 210 | 133.86 | 46.53 | Field surveys | <500m |
| 211 | 133.87 | 46.69 | Field surveys | <500m |
| 212 | 134.08 | 47.29 | Field surveys | <500m |
| 213 | 134.04 | 46.77 | Field surveys | <500m |
| 214 | 133.68 | 46.54 | Field surveys | <500m |
| 215 | 133.43 | 46.63 | Field surveys | <500m |
| 216 | 133.36 | 46.74 | Field surveys | <500m |
| 217 | 133.83 | 46.67 | Field surveys | <500m |
| 218 | 130.40 | 43.12 | Field surveys | <500m |
| 219 | 130.70 | 43.02 | Field surveys | <500m |
| 220 | 130.55 | 43.06 | Field surveys | <500m |
| 221 | 130.93 | 43.10 | Field surveys | <500m |
| 222 | 130.88 | 43.01 | Field surveys | <500m |
| 223 | 131.04 | 43.03 | Field surveys | <500m |
| 224 | 131.07 | 42.96 | Field surveys | <500m |
| 225 | 131.09 | 43.10 | Field surveys | <500m |
| 226 | 131.18 | 43.41 | Field surveys | <500m |
| 227 | 130.85 | 42.90 | Field surveys | <500m |
| 228 | 130.96 | 43.14 | Field surveys | <500m |
| 229 | 129.90 | 43.51 | Field surveys | <500m |
| 230 | 129.53 | 43.42 | Field surveys | <500m |
| 231 | 121.97 | 53.39 | Field surveys | <500m |
| 232 | 121.92 | 53.32 | Field surveys | <500m |
| 233 | 128.02 | 46.69 | Field surveys | <500m |
| 234 | 128.10 | 46.60 | Field surveys | <500m |
| 235 | 127.95 | 46.60 | Field surveys | <500m |
| 236 | 129.22 | 47.22 | Field surveys | <500m |
| 237 | 129.54 | 47.07 | Field surveys | <500m |
| 238 | 129.77 | 47.48 | Field surveys | <500m |
| 239 | 129.04 | 47.68 | Field surveys | <500m |
| 240 | 129.45 | 47.76 | Field surveys | <500m |
| 241 | 129.27 | 47.78 | Field surveys | <500m |
| 242 | 129.37 | 47.91 | Field surveys | <500m |
| 243 | 128.71 | 47.47 | Field surveys | <500m |
| 244 | 128.73 | 47.55 | Field surveys | <500m |
| 245 | 129.54 | 48.18 | Field surveys | <500m |
| 246 | 130.06 | 48.40 | Field surveys | <500m |
| 247 | 129.97 | 48.50 | Field surveys | <500m |
| 248 | 129.91 | 48.48 | Field surveys | <500m |
| 249 | 129.99 | 48.53 | Field surveys | <500m |
| 250 | 130.04 | 48.33 | Field surveys | <500m |
| 251 | 129.73 | 48.35 | Field surveys | <500m |
| 252 | 129.80 | 48.36 | Field surveys | <500m |
| 253 | 129.89 | 48.18 | Field surveys | <500m |
| 254 | 129.95 | 48.22 | Field surveys | <500m |
| 255 | 129.96 | 48.06 | Field surveys | <500m |
| 256 | 130.04 | 48.11 | Field surveys | <500m |
| 257 | 129.87 | 48.65 | Field surveys | <500m |
| 258 | 129.97 | 48.68 | Field surveys | <500m |
| 259 | 129.77 | 48.66 | Field surveys | <500m |
| 260 | 121.74 | 50.90 | Field surveys | <500m |
| 261 | 134.55 | 48.40 | Field surveys | <500m |
| 262 | 134.46 | 48.40 | Field surveys | <500m |
| 263 | 134.66 | 48.38 | Field surveys | <500m |
| 264 | 134.09 | 48.15 | Field surveys | <500m |
| 265 | 122.83 | 52.41 | Field surveys | <500m |
| 266 | 126.57 | 50.17 | Field surveys | <500m |
| 267 | 128.14 | 49.36 | Field surveys | <500m |
| 268 | 128.89 | 48.77 | Field surveys | <500m |
| 269 | 127.07 | 48.57 | Field surveys | <500m |

Note: Field surveys: 98.14%; Literature and reports: 1.86%.

**Figure S1 The results of all four Mann-Whitney U tests**

**
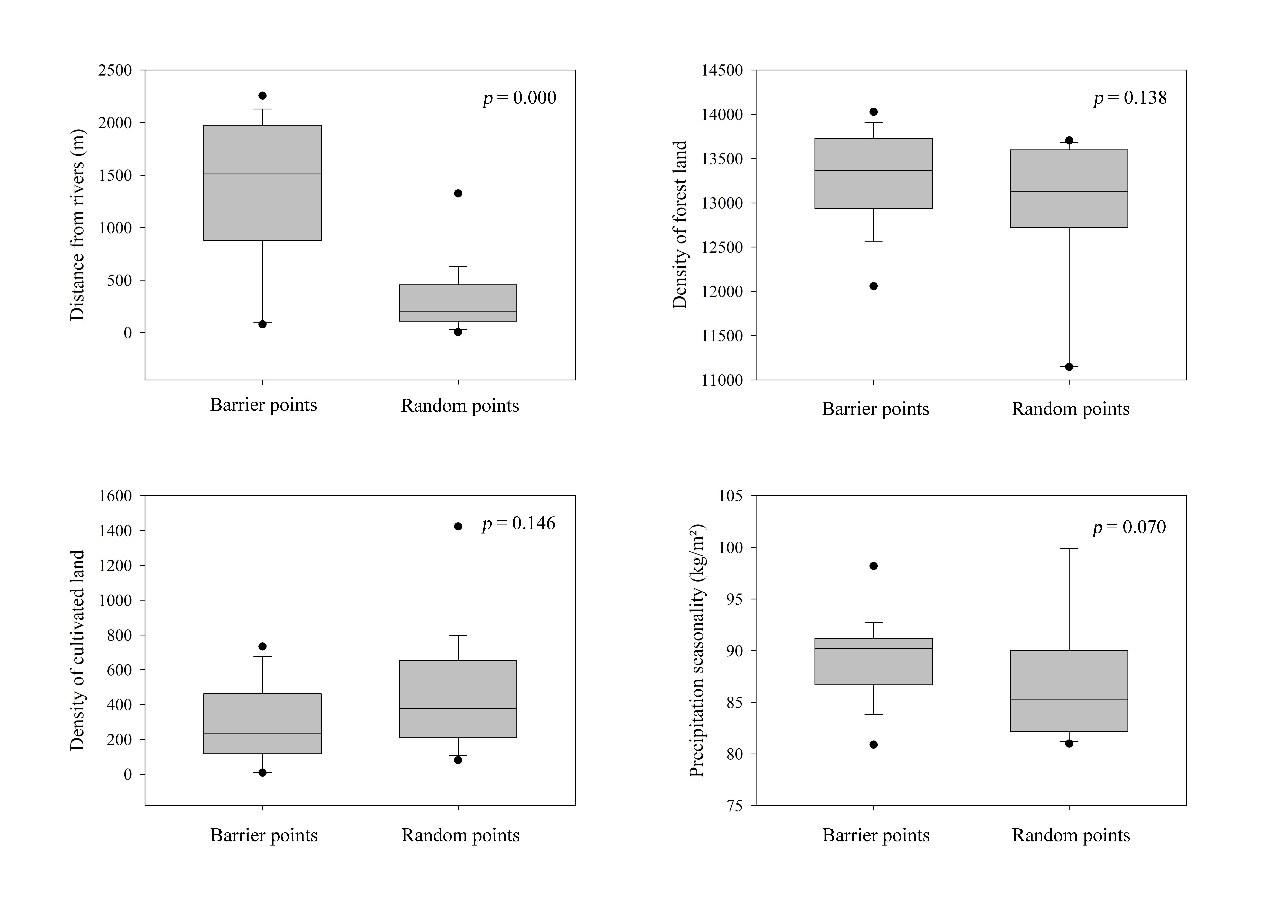
**

**Code S1 The complete analysis code**

**# Data preprocessing**

library(usdm)

path<-("…environmental variables…")

files_list<-list.files(path,pattern='.tif$',all.files = T,full.names = T)

files_list

multi_layer_spatraster<-rast(files_list)

multi_layer_spatraster

vif(multi_layer_spatraster)

**# Generation of pseudo-absence points**

library(biomod2)

library(terra)

DataSpecies<-read.csv("…occurrence…")

head(DataSpecies)

myRespName<-'presence'

myResp<-as.numeric(DataSpecies[,myRespName])

myRespXY<-DataSpecies[,c("X","Y")]

path<-("…environmental variables…")

mylist<-list.files(path,pattern='.tif$',all.files = T,full.names = T)

myExpl<-terra::rast(mylist)

myBiomodData<-BIOMOD_FormatingData(resp.var = myResp,

expl.var = myExpl,

resp.xy = myRespXY,

resp.name = myRespName,

PA.nb.rep = 3,

PA.nb.absences = 269,

PA.strategy = 'random',

na.rm = F)

myBiomodData

summary(myBiomodData)

plot(myBiomodData)

**# Implementation of the models**

myBiomodModelOut<-BIOMOD_Modeling(bm.format = myBiomodData,

modeling.id = 'AllModels',

models = c('RF','XGBOOST'),

prevalence = 0.5,

CV.strategy = 'random',

CV.nb.rep = 10,

CV.perc = 0.8,

metric.eval = c('TSS','ROC'))

myBiomodModelOut

**# Model validation procedures**

myBiomodEM <- BIOMOD_EnsembleModeling(bm.mod = myBiomodModelOut,

models.chosen = 'all',

em.by = 'all',

em.algo = c('EMwmean'),

metric.eval = c('TSS', 'ROC'),

var.import = 3,

seed.val = 42)

myBiomodEM

get_evaluations(myBiomodEM)

options(max.print=1000000)

get_variables_importance(myBiomodEM)

**# Projection of models**

myBiomodEMProj <- BIOMOD_EnsembleForecasting(bm.em = myBiomodEM,

proj.name = 'CurrentEM',

new.env = myExpl,

models.chosen = 'all',

metric.binary = 'all',

metric.filter = 'all')

myBiomodEMProj

plot(myBiomodEMProj)
